# Inducible transgene expression in PDX models in vivo identifies KLF4 as a therapeutic target for B-ALL

**DOI:** 10.1186/s40364-020-00226-z

**Published:** 2020-09-16

**Authors:** Wen-Hsin Liu, Paulina Mrozek-Gorska, Anna-Katharina Wirth, Tobias Herold, Larissa Schwarzkopf, Dagmar Pich, Kerstin Völse, M. Camila Melo-Narváez, Michela Carlet, Wolfgang Hammerschmidt, Irmela Jeremias

**Affiliations:** 1 Research Unit Apoptosis in Hematopoietic Stem Cells, Helmholtz Zentrum München, German Research Center for Environmental Health (HMGU), Marchioninistraße 25, 81377 Munich, Germany; 2 Research Unit Gene Vectors, Helmholtz Zentrum München, German Research Center for Environmental Health (HMGU), Munich, Germany; 3 Laboratory for Leukemia Diagnostics, Department of Medicine III, University Hospital, LMU Munich, Munich, Germany; 4 German Center for Infection Research (DZIF), Partner Site Munich, Munich, Germany; 5 German Cancer Consortium (DKTK), Partner Site Munich, Munich, Germany; 6 Department of Pediatrics, Dr. von Hauner Children’s Hospital, Ludwig Maximilian University, Munich, Germany

**Keywords:** PDX models of acute leukemia, Inducible transgene expression, KLF4, Azacitidine

## Abstract

**Background:**

Clinically relevant methods are not available that prioritize and validate potential therapeutic targets for individual tumors, from the vast amount of tumor descriptive expression data.

**Methods:**

We established inducible transgene expression in clinically relevant patient-derived xenograft (PDX) models in vivo to fill this gap.

**Results:**

With this technique at hand, we analyzed the role of the transcription factor Krüppel-like factor 4 (KLF4) in B-cell acute lymphoblastic leukemia (B-ALL) PDX models at different disease stages. In competitive preclinical in vivo trials, we found that re-expression of wild type KLF4 reduced the leukemia load in PDX models of B-ALL, with the strongest effects being observed after conventional chemotherapy in minimal residual disease (MRD). A nonfunctional KLF4 mutant had no effect on this model. The re-expression of KLF4 sensitized tumor cells in the PDX model towards systemic chemotherapy in vivo. It is of major translational relevance that azacitidine upregulated KLF4 levels in the PDX model and a KLF4 knockout reduced azacitidine-induced cell death, suggesting that azacitidine can regulate KLF4 re-expression. These results support the application of azacitidine in patients with B-ALL as a therapeutic option to regulate KLF4.

**Conclusion:**

Genetic engineering of PDX models allows the examination of the function of dysregulated genes like KLF4 in a highly clinically relevant translational context, and it also enables the selection of therapeutic targets in individual tumors and links their functions to clinically available drugs, which will facilitate personalized treatment in the future.

## Introduction

Tumor cells are characterized by multiple alterations at mRNA and protein levels. While nongenomic alterations are easily identified by techniques such as RNA sequencing or proteomics [1–3], the functional consequences of these alterations are generally uncertain, and only a small minority of such alterations might play an essential role in the growth and maintenance of the tumor. Identifying essential alterations is of major clinical importance because they represent putative targets for treatment [1–4]. Clearly, techniques to determine the functional consequences of alterations are lacking, but we established a clinically relevant method to identify alterations with life-sustaining function in individual tumors in vivo.

As a model system, we chose patient-derived xenografts (PDX), which avoids many of the limitations associated with the use of conventional cell line models or primary patient tumor cells [5–7]. PDX faithfully recapitulate the genetic and phenotypic characteristics of their parental tumors and their preclinical value for drug testing and biomarker identification is well established [8, 9]. Thus, the use of orthotopic PDX models represent the most clinically relevant approach to date for studying individual tumors [6, 10]. We studied B-cell precursor ALL (B-ALL), the most common malignancy in children which displays a favorable prognosis at first diagnosis due to conventional chemotherapy and, e.g., CAR-T-cell therapy, but harbors an urgent need for better treatment options upon prognostically challenging disease relapse [11].

To mimic the situation faced by patients, established tumors should already exist in the preclinical surrogate animals at the moment of molecular manipulation, which requires the use of inducible in vivo expression systems. While these techniques are well established in most other tumor models and normal human lymphocytes, PDX models are often excluded from functional genomic studies [12], and inducible orthotopic PDX models have not been reported.

We generated an inducible expression system in PDX models in mice in vivo. We applied and tested the method using the signaling molecule and transcription factor KLF4, which is implicated in the stress-responsive regulation of cell cycle progression, apoptosis and differentiation as well as stemness and pluripotency [13–19]. KLF4 is downregulated in numerous cancers but upregulated in others, and abnormal KLF4 expression might reflect oncogenic or tumor suppressor functions depending on the cellular context, tumor type, subtype and stage [14, 17, 20–27]. KLF4 is frequently deregulated in T-ALL and B-cell tumors [28–34], and is reportedly downregulated in pediatric B-ALL [35–38]. While KLF4 downregulation has been implicated in B-ALL leukemogenesis [39], its functional role in established B-ALL cells from patients in vivo is uncertain.

We developed a tetracycline-inducible expression system to re-express KLF4 in PDX B-ALL cells in vivo*.* We demonstrate here that KLF4 expression reduces tumor growth and enhances the chemotherapeutic response in this tumor model. With the aid of a CRISPR/Cas9-mediated KLF4 knockout in PDX cells, we further demonstrated that azacitidine exerts its antitumor effect by upregulating KLF4, supporting our interpretation. Our data demonstrate that inducible gene expression in PDX models is feasible and can be used to characterize the contribution of selected genes to tumor maintenance and to obtain valuable information regarding therapy responses. Our results reveal that KLF4 is a therapeutic target of interest in B-ALL, supporting the use of KLF4-regulating drugs in clinical trials of B-ALL.

## Materials and methods

### Ethical statements

Prior to obtaining the two primary B-ALL patient samples (Table S1), written informed consent was obtained from all patients or from parents/caregivers in cases in which patients were minors. The study was performed in accordance with the ethical standards of the responsible committee for human experimentation (written approval by the Ethikkommission des Klinikums der Ludwig-Maximilians-Universität Munich, number 068–08 and 222–10) and with the Helsinki Declaration of 1975, as revised in 2000.

Animal trials were performed in accordance with the current ethical standards of the official committee on animal experimentation (written approval by the Regierung von Oberbayern, tierversuche@reg-ob.bayern.de; July 2010, number 55.2–1-54-2531-95-10; July 2010, 55.2–1-54-2531.6-10-10; January 2016, ROB-55.2 Vet-2532. Vet_02–15-193; May 2016, ROB-55.2 Vet-2532. Vet_02–16-7 and August 2016, ROB-55.2 Vet-2532.Vet_03–16-56).

### Genetic engineering of EBV

In the maxi-Epstein Barr virus (EBV) plasmid, wtKLF4 and mutKLF4 cDNAs were fused to the 3′ open reading frame of the viral EBNA2 gene via a T2A element, which mediated the coexpression of both genes from the same transcript. While the wtKLF4 construct contained the entire open reading frame, the mutKLF4 construct lacked the two N-terminal zinc finger domains [40]. Details on the generation of both mutant EBV constructs are available in the supplement.

### Genetic engineering of PDX B-ALL cells for inducible transgene expression

Primary patient B-ALL cells were transplanted into immunocompromised mice to generate the PDX models. PDX B-ALL cells were lentivirally transduced and transgenic cells were enriched using flow cytometry by gating on the recombinantly expressed fluorochromes as described previously [41]. For inducible transgene expression, PDX B-ALL cells were consecutively lentivirally transduced with three constructs containing the tet activator, the tet repressor and KLF4 expression cassettes under the control of the TRE promoter [42].

### In vivo experiments

Leukemia growth and treatment effects were monitored using bioluminescence in vivo imaging as described previously [41]. Competitive experiments were performed by mixing two derivate cell populations, each of which expressed a different transgene and distinct fluorochrome marker, and injecting both into the same animal. Human PDX cells were isolated and enriched from murine bone marrow or spleen as described previously [43] and the distribution of each subpopulation was measured by flow cytometry using the different recombinantly expressed fluorochrome markers.

### Protein expression analysis

Flow cytometry-enriched cell populations were incubated in lysis buffer (#9803, Cell Signaling Technology, Boston, USA) on ice for 30 min. Protein concentration was measured by BCA assay (#7780, New England Biolabs, Beverly, USA) and abundance of specific proteins determined by two different methods.

#### Conventional Western blotting

Equal amounts of protein were separated using 12% SDS-PAGE gels and transferred to a nitrocellulose membrane (TransBlott, Bio-Rad, Munich, Germany), incubated with antibodies against Caspase3 or PARP, washed and incubated with a secondary antibody coupled to an enzyme (GE Healthcare, Menlo Park, USA). Enyzme activity was visualized using a chemiluminescent substrate (Thermo scientific, USA).

#### Capillary protein immunoassay

The technology (WES, Simple Western, ProteinSimple, San Jose, USA) enables a Western-Blot-like analysis with the advantage of requiring only low cell numbers, typically 2.5 μg per lane. It was used as only minor cell numbers can be re-isolated from each mouse.

Procedures were performed following manufacturer’s instructions; in brief, each capillary was loaded with protein lysate and electrophoresis was performed; capillaries were processed to attach all proteins to the capillary wall and incubated with a single antibody; results were measured as emission curves from each capillary using the Compass software (ProteinSimple), including quantification. Due to very high sensitivity, equal loading is hard to achieve, but also not required, as protein amounts are calculated with high sensitivity and reliability.

Primary antibody against KLF4 was purchased from R&D systems (AF3640, Minneapolis, MN, USA), against PARP from Cell Signaling Technology (9542 T, USA), against GAPDH from R&D systems (MAB5718-SP, USA), against Caspase-3 from Millipore (05–654, Billerica, USA) and against β-Actin from Novus biologicals (NB600-501SS, Littleton, USA). A comparison of the results obtained using conventional Western Blot versus the capillary immunoassay is provided in Supplementary Figure 1.

### Methods described in the supplement

Details are provided in the supplement regarding transcriptome analysis (Table S2), the constructs used for the inducible expression of KLF4 and CRISPR/Cas9-mediated knockout of KLF4 (Table S3), cell isolation and culture conditions, EBV infection, in vitro culture, in vivo growth of PDX-ALL, monitoring of tumor burden and drug treatments, PDX cell homing assay, in vitro culture and drug treatment of cells, cell cycle analysis, RT-PCR (Table S4) and statistical analysis.

## Results

We focused on studying KLF4 as the primary example because it is frequently de-regulated in B-cell tumors [28–38], but functional in vivo data in advanced B-ALL disease is missing. We performed reverse genetics to decipher the role of KLF4 in certain B-cell tumors. To examine both tumor formation and tumor maintenance, we began with the in vitro surrogate model of EBV-induced B-cell transformation.

### KLF4 blocks EBV-induced transformation of B-cells in vitro

**Fig. 1:**
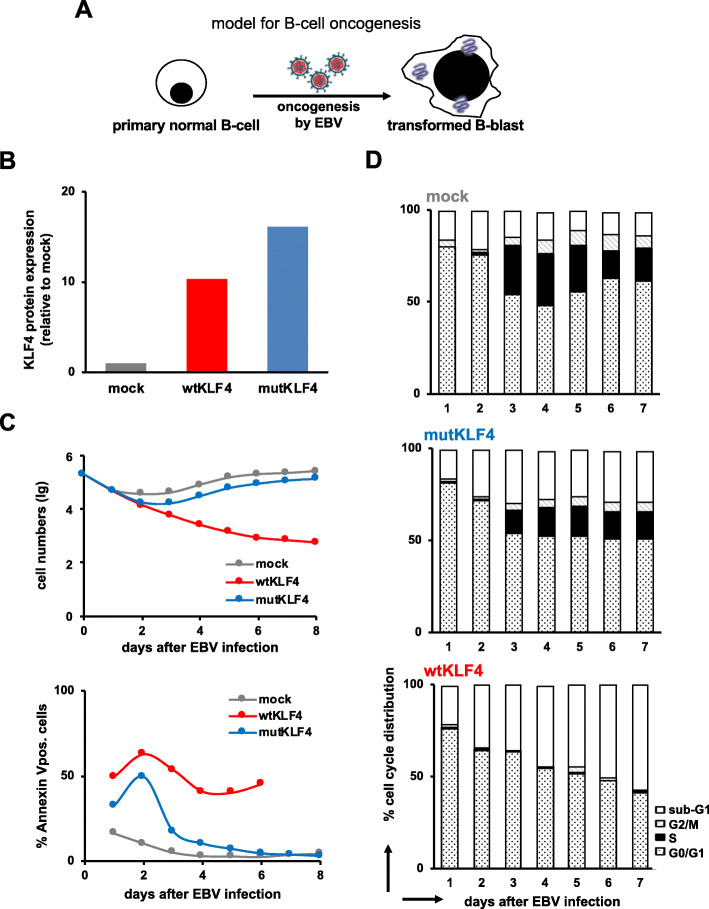
Ectopic expression of KLF4 blocks EBV-mediated oncogenesis. A total of 2 × 10^5^ normal naïve primary B-cells were infected with EBV in vitro to induce their transformation. B-cells were infected with a wildtype EBV strain (r_wt/B95.8 (6008), which contains all viral genes required for oncogenesis (mock) or with mutant EBV derivatives that additionally expressed either wildtype KLF4 (wtKLF4; 6948_EBNA2-T2A-KLF4) or a mutant derivative of KLF4 lacking its DNA-binding domain (mutKLF4; 6949_EBNA2-T2A-mutKLF4). After EBV infection, B-cells were harvested daily for up to 7 days. **a** Experimental design. **b** Capillary immunoassay of KLF4 2 days after infection with the indicated EBV viruses. β-Actin was used as a loading control. KLF4 expression was normalized to the loading control and was plotted as the fold change relative to that of the mock control. Representative result from two independent experiments are shown. **c** Cell numbers were quantified by flow cytometry (upper panel) and the fraction of apoptotic Annexin V binding cells was recorded (lower panel). The figure shows one experiment our of 3 replicates with B-lymphocytes obtained from three donors. **d** Cell cycle analysis: cells were incubated with 5-bromo-2′-deoxyuridine (BrdU) for 1 h prior to harvest and analyzed by flow cytometry after permeabilization and staining with a BrdU-specific antibody. The percentage of cells in the different phases of the cell cycle is indicated. See Supplementary Figure S3 for additional data

We chose this in vitro model of B-cell oncogenesis, which reprograms B-lymphocytes into B-blasts (Fig. 1a) [44–46] because we had previously detected, strong and persistent downregulation of *KLF4* mRNA levels during EBV-induced B-cell transformation (Supplementary Figure S2A and ref. [47]).

Two different variants of KLF4, wildtype KLF4 (wtKLF4) and a variant form of KLF4 (mutKLF4) were cloned into recombinant viruses (Supplementary Figure S2B and C). Upon infection, they coexpressed wtKLF4 or mutKLF4 together with viral genes in primary B lymphocytes. mutKLF4 lacks the two C-terminal zinc fingers representing the DNA binding domain, which disables the basic functions of KLF4 as a transcription factor, but the protein-protein interactions of KLF4 are not affected (Supplementary Figure S2B).

Naïve human B-lymphocytes were infected with EBV to induce their transformation into B-blasts by viral oncogenes (Fig. 1a, mock). To study the influence of KLF4 for the process of transformation, EBV was genetically modified and used as a shuttle vector to express wtKLF4 or mutKLF4 together with viral oncogenes in resting B-lymphocytes that undergo EBV-induced oncogenesis (Fig. 1b, Supplementary Figures S2C, 3A).

While mutKLF4 expression showed no major effects in EBV-infected B-cells compared with mock-EBV infection, the maintenance of wtKLF4 levels completely abrogated EBV-induced B-cell transformation, reduced EBV-induced B-cell proliferation and induced apoptosis in the infected cells (Fig. 1c, Supplementary Figure S3B). Cell cycle analysis indicated that KLF4 expression prevented S-phase entry in EBV-infected B-lymphocytes, which is required for the generation and expansion of B-blasts. Thus, KLF4 acts as a cell cycle inhibitor in the model of in vitro EBV-induced oncogenesis and conversion of normal B-cells into B-blasts.

In conclusion, persistent expression of KLF4 blocks human B-cell transformation in a model of EBV-induced oncogenesis.

This finding is in line with published data that suggest a role of KLF4 as tumor suppressor in BCR-ABL-mediated transformation of murine pre-B-cells into B-ALL [39]. Based on these findings and in line with our previous observation and published results that i) *KLF4* is severely downregulated in primary pediatric B-ALL cells [35–38], especially after therapy [43] (Supplementary Figure S4A), and ii) among adult ALL samples, the aggressive Ph^+^-like B-ALL subgroup showed low expression levels of KLF4 similar to those of T-ALL [22] (Supplementary Figure S4B); we went on to investigate a putative role of KLF4 in leukemia models of advanced disease.

### Inducible transgene expression in PDX cells in vivo

**Fig. 2:**
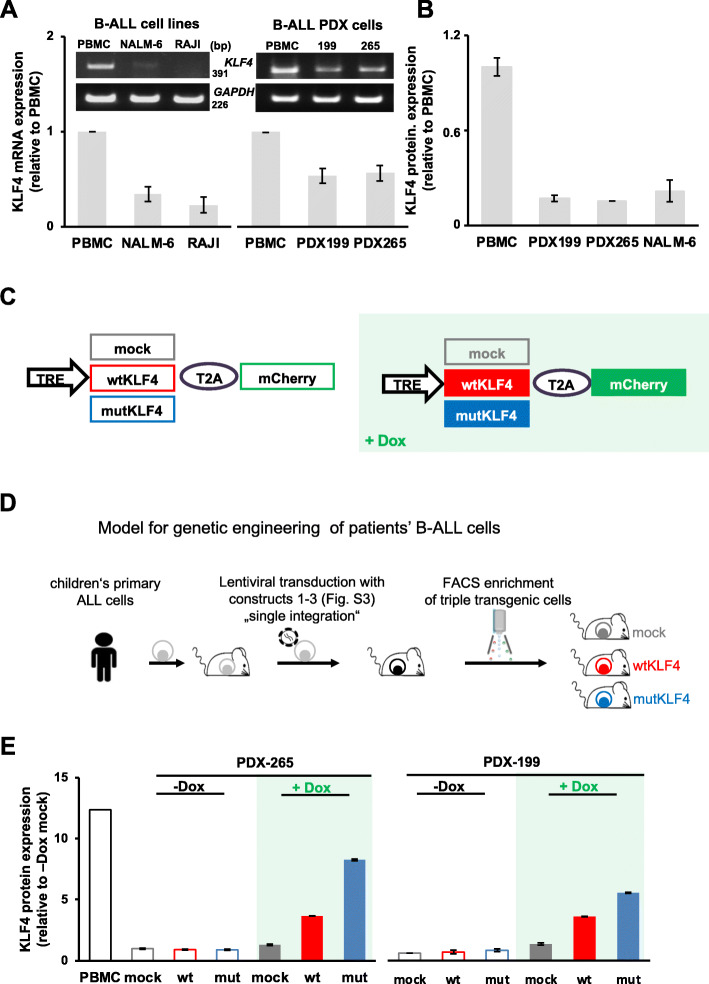
Re-expression of KLF4 in PDX ALL cells using a tet-on inducible system. **a**, **b** KLF4 is downregulated in B-ALL cell lines, B-cell lymphoma cell lines and B-ALL PDX cells as compared to peripheral blood mononuclear cells (PBMC). **a** *KLF4* mRNA was determined by RT-PCR using GAPDH as loading control; quantification of bands comparing KLF4 to GADH is depicted in the bar plot below. **b** KLF4 protein levels were analyzed by capillary immunoassay using β-Actin as loading control. KLF4 expression was normalized to the loading control and was plotted as the fold change relative to expression in PBMC. Mean ± SD of 3 independent experiments is shown. **c** KLF4 expression vector. The TRE promoter drives the expression of KLF4, which was either the wild-type (wt)KLF4 or a mutated (mut)KLF4 sequence lacking the two C-terminal zinc finger motifs comprising the DNA-binding domain (see Supplementary Figure S2B) that are each linked by a T2A peptide to the fluorochrome mCherry as a molecular marker; a mock vector (empty vector encoding only the mCherry fluorochrome) was used as a control. The addition of doxycycline (Dox; light green background throughout all figures) leads to the expression of the mock (gray), wtKLF4 (red) or mutKLF4 gene (blue; colors are identical throughout all figures) and mCherry (green). **d** Experimental design: Primary B-ALL cells from patients were transplanted into immunocompromised mice to generate the PDX models; PDX cells were consecutively transduced with three lentiviral constructs to express rtTA3 together with luciferase, tetR and either the mock, wtKLF4 or mutKLF4 gene (the vectors are detailed in panel C and Supplementary Figure S5A). Following passaging through mice for amplification, transgenic PDX cells were enriched by flow cytometry based on the constitutively expressed fluorochromes (mTaqBFP for the rtTA3-luciferase construct, iRFP720 or T-Sapphire for the tetR construct and Venus for the KLF4 constructs). Triple-transgenic cells were used for all further in vivo experiments. **e** PDX ALL-265 and PDX ALL-199 cells were infected as indicated in (**d**) and were cultured in vitro with or without the addition of Dox for 48 h, and KLF4 protein expression was analyzed by capillary immunoassays using PBMC from healthy donors as controls. β-Actin was used as a loading control. KLF4 expression is normalized to that of β-actin and is plotted as the fold change relative to the respective mock control. Mean ± SD of 3 independent experiments is shown

We asked whether KLF4 is functionally relevant for established B-cell tumors in vivo and whether it might represent a putative therapeutic target. As a clinically relevant model for functional genomic studies of established tumors, we used orthotopic PDX obtained from two children with relapsed B-cell precursor ALL (clinical data for both patients are listed in Supplementary Table S1). Similar to pediatric [35–38] and adult (Supplementary Figure S4B) primary B-ALL samples, both PDX B-ALL models revealed low KLF4 mRNA and protein levels (Fig. 2a and b).

For the conditional expression of KLF4 in PDX B-ALL samples, we engineered a tetracycline-response system to study the characteristics of PDX models independently of tumor cell transplantation and at different time points, e.g., before, during and after treatment and at the minimal residual disease stage.

PDX cells were consecutively transduced with three lentiviral constructs (Fig. 2c and d and Supplementary Figure S5A) to express (i) the tet activator (rtTA3) together with luciferase to allow in vivo bioluminescence imaging; (ii) the tet repressor (tetR) together with one of two different fluorochromes as molecular markers for competitive in vivo assays; and (iii) a mock control, wtKLF4 or mutKLF4 gene (Supplementary Figure S2B) in conjunction with the fluorochrome mCherry and under the control of the tet-responsive element (TRE) [42] (Fig. 2d and Supplementary Figure S5A). We selected a low transduction rate of 1% for each vector to permit the integration of only a single copy of each transgene per cellular genome. Although silencing of the CMV promoter is common in leukemic cells, the TRE-regulated mini-CMV promoter was not epigenetically repressed in our PDX models (data not shown). Doxycycline (Dox) treatment (indicated by the green background in all figures) of transduced B-ALL cells induced the expression of KLF4 together with mCherry, which serves as a proxy to monitor the coexpression of the wtKLF4 or mutKLF4 protein (Fig. 2e and Supplementary Figure S5B and C). Upon Dox withdrawal, the expression of mCherry and KLF4 returned to baseline levels, demonstrating the reversibility of the system (Supplementary Figure S5C). Notably, KLF4 protein expression in the induced state was moderate and did not exceed the levels observed in PBMCs, avoiding undesirable overexpression (Fig. 2e). Importantly, feeding Dox to mice induced the expression of mCherry/KLF4 in PDX B-ALL cells in vivo (Supplementary Figure S5B), enabling the analysis of KLF4-mediated effects at any given time point in mice. The combination of different marker fluorochromes provided the opportunity to investigate PDX cells with conditional wtKLF4 and control (mock) alleles in the same animal by pairwise competition experiments, abrogating animal-to-animal variability.

Taken together, the results showed that the newly established inducible system of transgene expression in PDX of acute leukemias appeared to be functional and represent an attractive method to identify and study leukemia vulnerabilities in individuals and to test established and potential drugs for their anti-leukemic potency in vivo*.*

### Re-expressing wtKLF4 reduces the growth and homing of B-ALL PDX cells in vivo

**Fig. 3:**
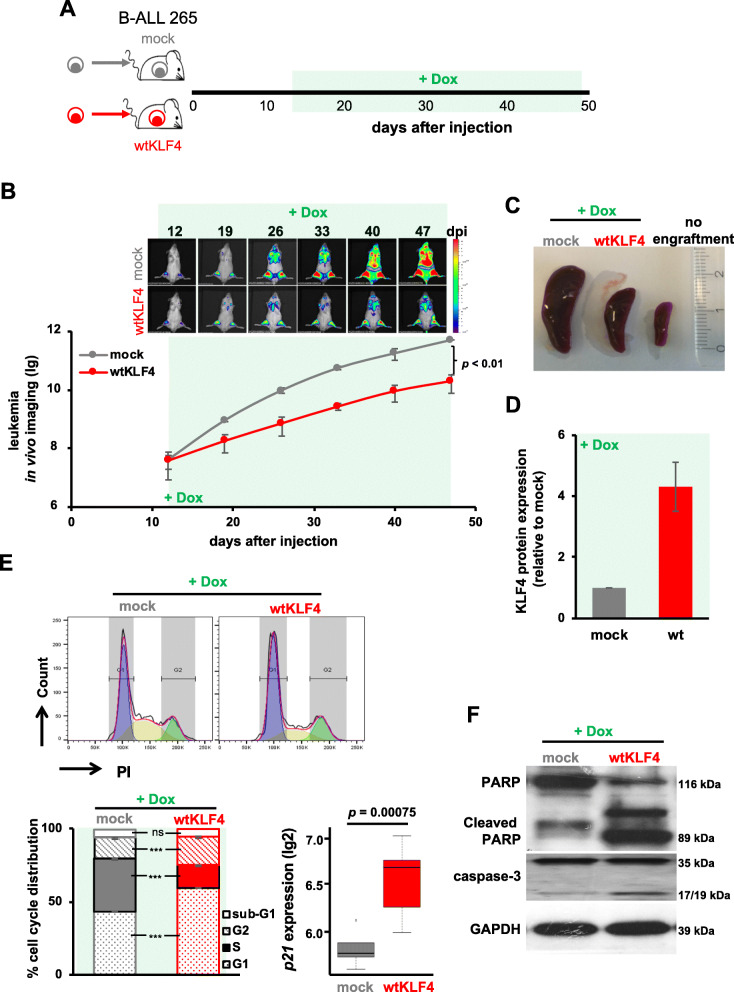
Re-expressing KLF4 inhibits B-ALL PDX growth through cell cycle arrest and apoptosis in vivo*.* **a** Experimental design: Sixty thousand triple-transgenic, mock or wtKLF4 PDX ALL-265 cells were injected into the tail vein of 6 NSG mice each. After homing was completed and the tumors were established, Dox (1 mg/ml) was added to the drinking water from day 12 onwards to induce KLF4 expression. On day 47 after cell injection, the mice were sacrificed, spleens harvested and the Dox-induced, mCherry-positive mock- or wtKLF4-expressing cell population was enriched by flow cytometry for further analysis. **b** In vivo bioluminescence imaging is shown as representative images (upper panel), and after the quantification of all 6 mice, the results are depicted as the mean ± SEM. *p* < 0.01 according to a two-tailed unpaired t test. **c** Representative spleens of mice, with a healthy mouse without leukemic engraftment for comparison. **d** The KLF4 protein level of mCherry-positive splenic cells was analyzed by capillary immunoassays; β-Actin served as the loading control. The mean ± SD of KLF4 expression normalized to that of β-Actin for 3 mice is plotted as the fold change relative to the mock control. **e** Cell cycle analysis: 10^6^ mCherry positive cells were stained with propidium iodide (PI) and cell cycle distribution was measured by flow cytometry. Upper panel: representative histograms of mock- (*n* = 3) or wtKLF4-expressing (*n* = 3) cells; lower panel: quantification of the mean ± SD is shown. *** *p* < 0.005 by two-tailed unpaired t test; ns = not significant. Lower right: Levels of p21 mRNA as determined by scrb sequencing. **f** PARP- and caspase-3 cleavage in mock- (*n* = 3) or wtKLF4-transduced (*n* = 3) PDX ALL-265 cells as determined by Western blotting; GAPDH was used as a loading control

**Fig. 4:**
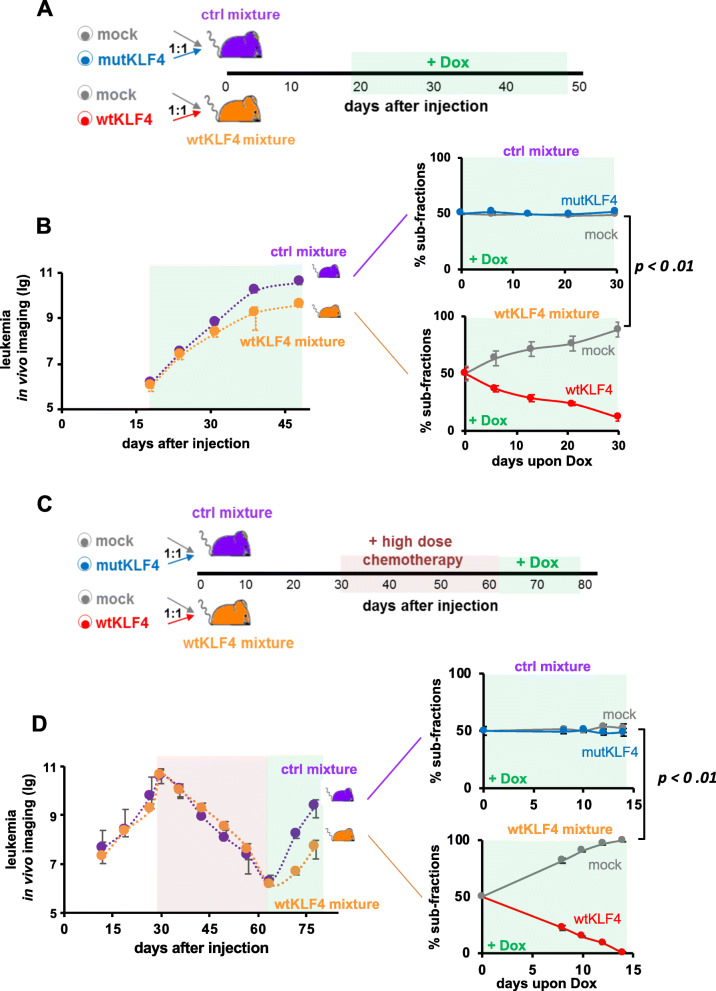
KLF4 re-expressing cells are outcompeted in competitive in vivo assays, especially after treatment. **a** Experimental procedure: NSG mice were injected with 30,000 cells from the control mixture (purple; *n* = 10) or wtKLF4 mixture (orange, *n* = 10). The control mixture consisted of a 1:1 ratio of mock- and mutKLF4-expressing cells; the wtKLF4 mixture consisted of a 1:1 mixture of mock- and wtKLF4-expressing cells. After homing was completed and the tumors were established, Dox was added to the drinking water from day 18 onward, and leukemia growth was monitored by in vivo bioluminescence imaging. Two mice from each group were sacrificed every week, and the proportions of the two subfractions in bone marrow were analyzed by flow cytometry. **b** Left panel: Leukemia growth as determined by in vivo imaging in mice bearing the wtKLF4 or control mixture. Right panel: Subfraction analysis by flow cytometry by gating on the subpopulation-specific fluorochrome markers T-Sapphire for the mock subpopulation and iRFP720 for either the mutKLF4 or wtKLF4 subpopulation (see Figure S2B for information about the constructs). Quantification is depicted as mean ± SEM. *p* < 0.01 according to a two-tailed unpaired t test. **c** Experiments were set up as described in **A**, except that Dox was administered after treatment, during tumor regrowth. When a high tumor burden was reached (day 31), mice were treated intravenously with high-dose combination chemotherapy (0.25 mg/kg vincristine + 100 mg/kg cyclophosphamide once per week, given on Mondays and Thursdays, respectively, light red background) to reduce the tumor burden to achieve minimal residual disease (MRD); at MRD (day 64), chemotherapy was stopped, and Dox was added to the drinking water to induce transgene expression. **d** Tumor regrowth after treatment was monitored and analyzed as described in (**b**)

**Fig. 5:**
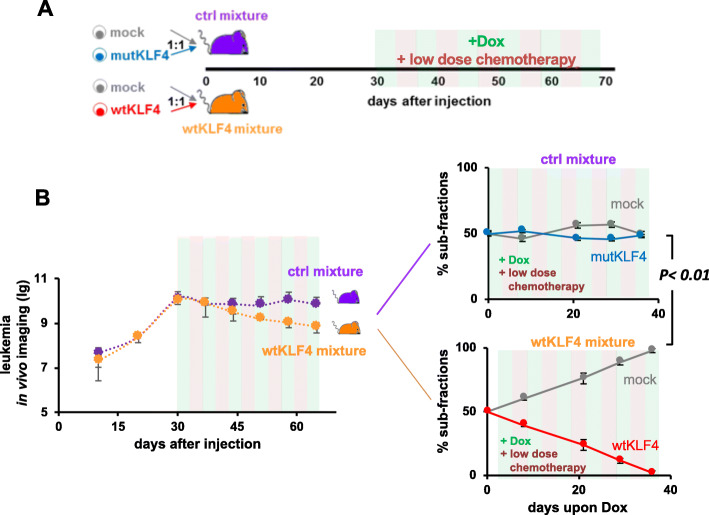
KLF4 expression sensitizes PDX ALL cells to chemotherapy. **a** Experiments were set up as shown in Fig. 3, except that transgene expression was initiated when a high tumor burden was achieved (day 30) and maintained during low-dose combination chemotherapy (0.2 mg/kg vincristine + 35 mg/kg cyclophosphamide; days 31–65). Dox was added to the drinking water starting on day 30 when starting the low dose combination chemotherapy to initiate transgene expression (light red- and light green-striped background). **b** Tumor growth was monitored and analyzed as shown in Fig. 3

In the first step, we determined whether low KLF4 expression levels were essential and required for the survival and proliferation of B-ALL tumors in vivo. Two PDX B-ALL samples, consisting of ALL-265 (Figs. 3, 4 and 5) and ALL-199 (Supplementary Figure S6) cells, that were transduced with either mock or wtKLF4 alleles were transplanted into groups of mice. When cell homing and early engraftment were completed, the mice were fed with Dox to induce transgene expression during the exponential growth phase of the pre-established PDX B-ALL tumors (Fig. 3a and Supplementary Figure S6A).

Tumor growth of wtKLF4-expressing B-ALL PDX cells was reduced compared with that of control cells, as determined by in vivo imaging (Fig. 3b and Supplementary Figure S6B). Consequently, KLF4 re-expression resulted in a reduction in splenomegaly compared with that in mock gene-bearing mice (Fig. 3c, d and Supplementary Figures S1 and 6CD). KLF4 significantly reduced the fraction of cells in S-phase, accompanied and eventually caused by upregulation of the bona fide KLF4 target gene p21 (Figs. 3e and Supplementary Figure S6E and Table S2), but also induced cleavage of PARP and caspase-3 (Fig. 3f and Supplementary Figure S6F) indicating that KLF4 impaired tumor growth by inducing cell cycle arrest and apoptosis. Comparable results were obtained in a B-ALL cell line (Supplementary Figure S7) supporting our observations in vivo.

The effect of KLF4 on tumor growth was further investigated in competition assays in vivo when identical numbers of mock-transduced control cells and wtKLF4-transduced cells derived from the same PDX model were mixed and injected into the same animal (Fig. 4a, indicated in orange). As an additional control, a second group of mice was injected with mock- and mutKLF4-transduced cells (indicated in purple in Fig. 4a). The leukemic load was monitored by in vivo imaging, and the cell ratios of the subpopulations were quantified using the respective fluorochromes.

In the competition experiments, mice injected with the wtKLF4 mixture displayed reduced tumor growth in vivo, although 50% of cells in the wtKLF4 mixture were control cells (Fig. 4b, left panels). Cells transduced with the wtKLF4 allele had a severe growth disadvantage and nearly disappeared within 30 days (Fig. 4b, lower right panel) whereas mock-transduced cells expanded readily. In control mice, the ratio of the two subpopulations remained constant over time (Fig. 4b, upper right panel) suggesting that genetic manipulation per se did not affect the oncogenic properties of the leukemic PDX models.

The in vivo competitive assay (Fig. 4a and b) confirmed the growth-inhibitory effect of wtKLF4 that we observed in previous experiments (Fig. 3). Among the major advantages of competitive in vivo assays is that they can correct for high intermouse variation and have a high reliability and sensitivity, and thus they represent a powerful tool to identify, even moderate functional contributions to tumor maintenance in vivo.

Ectopic expression of KLF4 impaired B-ALL growth and survival in vivo, but KLF4 might also affect engraftment into the murine bone marrow. To investigate this possibility, we analyzed the capacity of KLF4-expressing PDX cells to home in on the murine bone marrow in retransplantation experiments. Mock-transduced or wtKLF4 or mutKLF4 expressing PDX cells were isolated from the first recipient mice that received Dox at an advanced disease stage (Supplementary Figure S8A). Equal numbers of cells were combined and retransplanted into second-generation mice and cell homing was assessed 3 days later (Supplementary Figure S8A). The ratio of transplanted mock versus mutKLF4-expressing cells was not altered in the bone marrow after 3 days, but in reisolated cell populations obtained from mice that had received mock- and wtKLF4-transduced cells, the abundance of the latter was strongly reduced (Supplementary Figure S8B), indicating that wtKLF4 expression impairs homing of PDX B-ALL cells in vivo.

Taken together, the results demonstrated that the re-expression of KLF4 reduced the overall fitness of PDX B-ALL cells in vivo. KLF4 functions as a cell cycle inhibitor and pro-apoptotic factor in PDX B-ALL resulting in decreased growth of established tumors and an impaired ability to infiltrate the murine bone marrow.

### Treatment-surviving cells are especially sensitive towards the re-expression of KLF4

In B-ALL, persistent minimal residual disease (MRD) is the most highly predictive factor for disease-free survival [11], emphasizing the need to target and eradicate MRD. The number of primary MRD cells isolated from each patient is small by definition, so elaborate in vivo models are necessary to functionally characterize the MRD cell subpopulation, which is a serious technical challenge [43]. The inducible expression system provides the means to characterize the role of individual genes in MRD.

To mimic MRD in mice, we titrated a combination of the routine drugs vincristine and cyclophosphamide at clinically relevant doses to effectively reduce the tumor burden by several orders of magnitude over 5 weeks. When MRD was achieved (defined as less than 1% leukemia cells in the bone marrow) chemotherapy was discontinued and Dox was administered to induce KLF4 expression during the phase of tumor regrowth (Fig. 4c).

The treatment responses of the control cells and wtKLF4 mixture were comparable, as indicated by in vivo imaging (Fig. 4d, left panel). In contrast, upon Dox administration in MRD, tumor regrowth was clearly diminished in mice carrying PDX cells transduced with the wtKLF4 mixture (Fig. 4d, left panel). Subfractional analysis revealed that the expression of wtKLF4 (but not mutKLF4) was reduced, and finally, even cellular regrowth was prevented in vivo, which depleted wtKLF4 cells to undetectable levels within 2 weeks (Fig. 4d, right panel). When comparing therapy-naïve, wtKLF4-expressing cells (Fig. 4b) with similar cells obtained after chemotherapy in MRD (Fig. 4d, lower right panel), KLF4 inhibited the growth of MRD cells more dramatically than that of therapy-naïve PDX B-ALL cells (Supplementary Figure S9). It thus appears that MRD cells are especially sensitive to KLF4 expression, suggesting that any regimen that induces KLF4 expression in MRD might be especially effective for tumor consolidation therapy.

### KLF4 sensitizes B-ALL PDX cells to chemotherapy in vivo

Low KLF4 expression levels were a prerequisite for the in vivo growth of PDX B-ALL samples, especially after mice underwent experimental chemotherapy. Our subsequent experiments addressed the question of whether the re-expression of KLF4 sensitizes patients’ B-ALL cells to conventional chemotherapy during routine ALL treatment in vivo in the setting of induction therapy.

PDX B-ALL control and wtKLF4 mixtures were injected into mice, and Dox was administered together with chemotherapy at a high tumor burden (Fig. 5a). In this experiment, Dox was administered simultaneously with chemotherapy, which was administered at a dose intended to stop tumor progression but not reduce the tumor size to facilitate the identification of KLF4-induced phenotypes.

Accordingly, chemotherapy prevented further tumor progression in mice injected with control cells as intended, but it also reduced the tumor load in mice injected with PDX cells transduced with control and wtKLF4-expressing vectors (Fig. 5b, left panel). When the subcellular fractions of these animals were analyzed, the number of wtKLF4- but not mutKLF4-expressing cells was significantly decreased by chemotherapy, and therefore the wtKLF4-expressing cells were outcompeted and lost within less than 40 days (Fig. 5b, right panel). KLF4 re-expression also sensitized NALM-6 cells to chemotherapy in vitro (Supplementary Figure S10) confirming the results obtained from PDX B-ALL cells in vivo. The data suggest that the upregulation of KLF4 may synergize with standard therapeutic regimens, i.e. conventional chemotherapy to eliminate B-ALL cells in patients.

### Azacitidine-induced cell death partially depends on KLF4

Next, we searched for drugs capable of upregulating KLF4. We first tested the small molecule APTO-253, which was developed as a KLF4-inducing drug [48] but was recently shown to also regulate MYC [49]. APTO-253 indeed upregulated KLF4 in our experiments and sensitized cell lines towards vincristine treatment in vitro (Supplementary Figure S11), indicating that KLF4 can, in principle, be reupregulated by drugs in B-ALL.

**Fig. 6:**
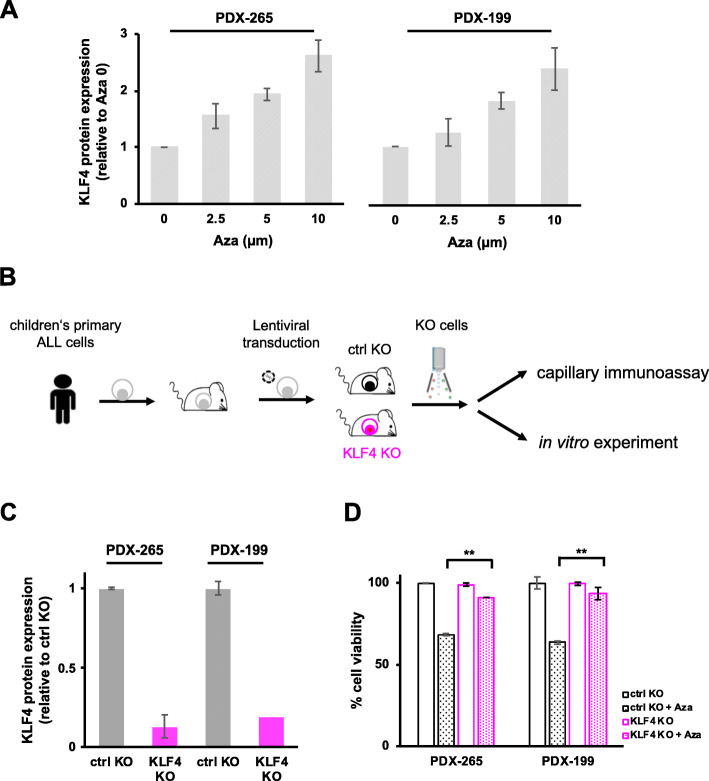
Azacitidine-induced cell death depends on KLF4. **a** PDX-265 and PDX-199 cells were treated with different concentrations of azacitidine (Aza) for 48 h. KLF4 protein expression was analyzed by capillary immunoassays, and β-Actin served as a loading control. One representative analysis of 2 independent experiments is shown. KLF4 expression normalized to β-Actin was plotted as fold change relative to the expression of respective untreated cells (Aza0). Mean ± SD of 3 independent experiments is shown. **b** Experimental procedure: B-ALL PDX cells were lentivirally transduced with Cas9, sgRNA and reporter expression vectors (Supplementary Figure S12B) and injected into NSG mice to generate ctrl KO (black) and KLF4 KO (pink) PDX cells. Mice were sacrificed once full blown leukemia had developed, and marker-positive populations were enriched by flow cytometry by gating on the recombinant markers (mTaqBFP for Cas9, mCherry for the sgRNAs and GFP for the reporter construct) and the cells were subjected to capillary immunoassays and in vitro culture. **c** KLF4 protein levels in ctrl KO or KLF4 KO PDX-265 and PDX-199 cells were analyzed by capillary immunoassays. Mean ± SEM for KLF4 expression according to 2 experiments normalized to ß-Actin is plotted as the fold change relative to the expression of the control KO cells. **d** PDX B-ALL ctrl KO cells and KLF4 KO cells were treated with 2.5 μM Aza in vitro for 48 h and cell viability was measured by flow cytometry. Viability was normalized to that of nontreated cells. The mean ± SEM of duplicates is shown. ** *p* < 0.01 by two-tailed unpaired t test

We speculated that existing approved drugs might be able to reupregulate KLF4 in B-ALL to facilitate clinical translation. The demethylating agent 5-azacitidine (Aza) was shown to upregulate KLF4 expression in other tumor entities [22, 28, 31, 50]. Although Aza can reverse DNA hypermethylation, it regulates protein expression on multiple levels, and multiple mechanisms have been shown to contribute to the antitumor activity of Aza [51, 52]. Aza is increasingly used in clinical trials on patients with hematopoietic malignancies and MRD, e.g., in acute myeloid leukemia, to prevent or delay relapse [53]. Here we found that, already within 48 h, Aza upregulated KLF4 levels in B-ALL PDX cells as well as B-ALL cell lines (Fig. 6a and Supplementary Figure S12A), and decreased cell viability in vitro at clinically relevant doses (Supplementary Figure S12A).

To assess whether Aza-induced cell death in B-cell tumor cells is mediated by increased levels of endogenous KLF4 protein, we generated KLF4 knockout (KO) cells, which is a challenge for PDX. For B-ALL PDX, cells were transduced with a lentiviral vector to introduce Cas9 (Fig. 6b and Supplementary Figure S12B). In subsequent steps, a pool of 3 vectors, each encoding a single KLF4 targeting guide RNA, and a reporter construct were introduced to enrich gene-edited cells. PDX B-ALL cells with stable KO of KLF4 could readily be established (Fig. 6c).

Stimulation of PDX B-ALL cells with Aza at clinically relevant concentrations reduced cell viability in control cells but not in PDX B-ALL cells with KLF4 KO (Fig. 6d). Similar results were obtained in three B-ALL cell lines (Supplementary Figure S13), using either individual sgRNAs or pools of 3 sgRNAs to generate KLF4 KO cells. These data suggest that KLF4 at least partially mediates the effect of Aza on B-ALL cells.

Taken together, the results indicate that KLF4 might represent a novel therapeutic target for B-ALL. Our data also show that Aza, an established drug, can upregulate KLF4. Introducing Aza into standard polychemotherapy protocols for B-ALL patients with the intention of raising KLF4 levels might reduce tumor burden and increase sensitivity to conventional chemotherapy. Patients with B-ALL might benefit from this treatment option; further investigation for proof-of-concept should be performed in clinical trials.

## Discussion

We established inducible transgene expression in preclinical PDX models in vivo that allow the characterization of alterations in tumor cells with functional relevance. Using this model, we demonstrate that the two highly aggressive PDX B-ALL models studied as well as several cell lines depend on downregulation of KLF4; reupregulating KLF4 using an inducible expression system or by treatment with Aza impairs tumor maintenance in vitro and in vivo. The limited number of models studied prevent drawing strong general conclusions; nevertheless, the two highly aggressive PDX B-ALL models and several cell lines studied showed identical phenotypes.

We work with genetically engineered PDX models that we propose to name “GEPDX models”, in accordance with the term genetically engineered mouse models (GEMM). Inducible GEMM were designed to allow the determination of the role of single molecules independently from gestation, for example. Similarly, our novel inducible GEPDX models allow for the determination of the role of single molecules in pre-established tumors independently of tumor transplantation into mice. Inducible GEPDX models might be established for a broad range of different tumor types in addition to B-ALL and show the potential to interrogate gene function in preclinical cancer models at clinically relevant time points, such as in MRD.

Using these approaches, we demonstrate that KLF4 i) impairs B-ALL maintenance in vivo*,* ii) strongly reduces the regrowth of B-ALL PDX cells in MRD, and iii) increases the response of PDX B-ALL cells to therapy. We show that the well-described roles of KLF4 as a quiescence factor and apoptosis inducer are maintained in the malignant B cells studied [14]. We show that Aza upregulates KLF4 in B-ALL, which mediates, at least in part, the anti-leukemia effect of Aza; this result is of major translational importance.

Aza has previously been shown to upregulate KLF4 in other tumor entities [22, 31, 54–56] and we demonstrate that this ability is maintained in B-ALL. A proven clinical benefit of Aza treatment for hematological malignancies was first observed in myelodysplastic syndrome and AML [51], without increasing the incidence of secondary malignancies. In B-ALL, case reports on the clinical activity of Aza [57–59] exist, and an ongoing clinical study applies Aza to the treatment of patients with B-ALL with KMT2A rearrangements (NCT02828358). As the molecular mechanism of Aza-mediated antitumor activity remains incompletely defined [51], our data suggest that KLF4 upregulation represents an important mechanism by which Aza eliminates B-ALL cells.

The data presented here highlight the power of molecular/functional testing to improve the understanding of the alterations in individual tumors and to link gene function to available drugs. As we discovered that downregulating KLF4 plays an essential role in the growth of 2 established PDX models of B-ALL in vivo and that the widely used drug Aza upregulates KLF4, our data support the further evaluation of Aza as a therapeutic strategy and the idea of applying Aza to the treatment of patients with B-ALL.

## Supplementary information

**Additional file 1.**

## Data Availability

All datasets used in this study are publicly available.

## Abbreviations

ALL: Acute lymphoblastic leukemia
AML: Acute myeloid leukemia
AZA: Azacitidine
B-ALL: B-cell ALL
CMV: Cytomegalovirus
CRISPR: Clustered Regularly Interspaced Short Palindromic Repeats
Dox: Doxycycline
EBV: Eppstein-Barr Virus
GEMM: Genetically engineered mouse models
GEPDX: Genetically engineered PDX models
LUC: Luciferase
MRD: Minimal residual disease
PBMC: Peripheral blood mononuclear cells
PDX: Patient-derived xenografts
T-ALL: T-cell ALL
Tet: tetracycline
TRE: Tet response element
