# Supplementary material for: Inducible transgene expression in PDX models in vivo identifies KLF4 as a therapeutic target for B-ALL
Source: Biomark Res. 2020 Sep 16;8:46. doi: 10.1186/s40364-020-00226-z (PMC7493381; doi:10.1186/s40364-020-00226-z)
Supplement: Supplementary file 1 — Additional file 1. [file 40364_2020_226_MOESM1_ESM.pdf]

Supplementary Information

Inducible transgene expression in PDX models *in vivo* identifies KLF4 as a therapeutic target for B-ALL

Wen-Hsin Liu[1], Paulina Mrozek-Gorska[2], Anna-Katharina Wirth[1], Tobias Herold [1,3,4], Dagmar Pich[2], Kerstin Völse[1], M. Camila Melo-Narváez[2], Michela Carlet[1], Wolfgang Hammerschmidt[2,5], Irmela Jeremias[1,4,6*]

<u>This file contains:</u>

Supplementary Methods

Supplementary References

Supplementary Tables

Supplementary Figures and Legends

Supplementary Methods

Constructs for inducible KLF4 expression and KLF4 knockout

Tet-on inducible expression system (1): The pRRL-SFFV-rtTA3-IRES-Luc2-P2A-mtagBFP was a gift from Johannes Zuber (Research Institute of Molecular Pathology, Vienna, Austria). The two DNA fragments that contained the coding sequences of tetracycline repressor protein (tetR), coupled via T2A either to iRPF720 or T-Sapphire were synthesized (IDT DNA technologies, Coralville, IA), and cloned into the pCDH vector (Addgene plasmid #104834) using BamHI and SalI. For KLF4 transgene expression, The wtKLF4-T2A-mCherry and mutKLF4-T2A-mCherry DNA fragments were synthetized (IDT DNA technologies), amplified, and cloned into the pRRL-TRE-mCherry-PGK-Venus vector (based on TRMPV, Addgene plasmid # 27991, but dsRED was replaced by mCherry) using BamHI and XhoI. mutKLF4 lacks the amino acids 462-513 (NP_001300981.1), encoding zinc finger domain 2 and 3 (2).

CRISPR/Cas9 expression vectors: A DNA fragment containing the cDNA of spleen focus forming virus (SFFV) promoter (Addgene plasmid #27348), humanized Staphyococcus pyogenes Cas9 (hSpCas9 PX330, Addgene plasmid #42230) and a T2A-linked mTaqBFP fluorochrome (pTagBFP-C vector, PF171, Envrogen, Lawrenceville, NJ) was amplified by PCR and cloned into the pCDH vector (Addgene Plasmid #72263) using the SpeI/EcoRI, EcoRI/BamHI and NsiI/SalI restriction sites, respectively. For sgRNA expression, the pCDH vector (Addgene Plasmid #104833) expressing mCherry under control of the Elongation factor 1-alpha (EF1α) promoter was complemented by an additional H1 promoter expressing the sgRNA; towards this aim, an oligonucleotide containing the H1 promoter, a BbsI cutting site (Addgene plasmid #42230), a gRNA scaffold sequence (Addgene plasmid #42230) and a polymerase III terminator sequence (gift from Macro J. Herold, Walter and Eliza Hall Institute, Australia) were ordered from IDT DNA technologies and ligated into the pCDH backbone between the cPPT and EF1α promoter using ClaI and SpeI. The sgRNA sequences (Table S3) were synthesized (Eurofins MWG-Operon, Ebersberg, Germany), annealed and ligated into the vector. A detailed description of the CRISPR/Cas9 reporter system used will be provided upon request.

EBV-induced transformation of normal naïve human B-cells

EBV constructs: The wild-type EBV strain, termed r_wt/B95.8 (6008) (3) is based on the maxi-EBV plasmid p2089, which comprises the entire B95.8 EBV genome cloned onto a mini-F-factor plasmid in *E. coli* (4). The EBV construct is a reconstructed EBV field strain that carries the right handed oriLyt and expresses all 25 EBV-encoded pri-miRNAs from their endogenous viral promoters as well as the LF1, LF2 and LF3 genes that are absent in the genomic DNA of the B95.8 EBV strain.

Two mutant EBV strains that express the *KLF4* cDNA (6948_EBNA2-T2A-KLF4; wtKLF4) and a DNA-binding deficient mutant KLF4 cDNA (6949_EBNA2-T2A-mutKLF4; mutKLF4 (2)) were constructed (Supplementary Figure 2C). wtKLF4 and mutKLF4 cDNAs were fused to the 3' open reading frame of the viral EBNA2 via a T2A element, mediating co-expression of both genes from the same transcript (Supplementary Figure 2C). Details of the genetic engineering of the two mutant EBVs and their DNA sequences have previously been published (3, 5) and further details are available upon request.

Isolation of naïve B-lymphocytes: Adenoid biopsies from children were mechanically disintegrated to obtain single cell suspensions. Human B lymphocytes were separated from T cells by rosetting with defibrinated sheep blood and purified by Ficoll–Hypaque (Biochrom AG, Berlin, Germany) density gradients centrifugation. The interphase with the B lymphocytes was removed and washed three times with PBS. The cells were counted and directly used for staining with antibodies against CD38 (#25-0389-42, eBioscience, San Diego, USA) and IgD (#555778, BD Pharmingen, San Diego, USA). Naïve B-lymphocytes (IgD^+/IgH^+ and $CD38^-$) were physically sorted with a BD FACSAria™ III instrument (Becton-Dickinson, San Jose, USA) and used for further experiments.

EBV infection and *in vitro* culture: Primary B cells were infected with mock-EBV (r_wt/B95.8 (6008)), wtKLF4-EBV (6948), and mutKLF4-EBV (6949) with a multiplicity of infection (MOI) of 0.1 overnight. The next day, the medium was replaced with fresh medium and the infected cells were seeded in 96-well cluster plates at an initial concentration of 10^6 cells/ml.

Cell isolation and *in vitro* culture conditions

<u>Cell culture conditions:</u> B-cells were cultivated in RPMI1640 medium supplemented with 8% FCS, 100 U/ml streptomycin/penicillin, 1 mM sodium pyruvate, 100 mM sodium selenite, and 0.43% α-thioglycerols. NALM-6 (human pre B-ALL) and RAJI (Burkitt lymphoma cell line, EBV-positive) were purchased from DSMZ (German collection of microorganisms and cells, Braunschweig, Germany) and were maintained in RPMI-1640 supplemented with 2 mM L-glutamine (Life Technologies GmbH, Darmstadt, Germany) and 10% FCS (Biochrom AG). HEK293T cells were maintained in DMEM (Life Technologies) supplemented with 2 mM L-glutamine and 10% FCS. PDX cells were cultured in RPMI medium supplemented with 20% FSC, 1% pen/strep, 1% gentamycin, 6 mg/l insulin, 3 mg/l transferrin, 4 µg/l selenium (Gibco, San Diego, USA), 2 mM glutamine, 1 mM sodium pyruvate, 50 µM α-thioglycerol (Sigma-Aldrich, St. Louis, USA).

<u>Peripheral blood mononuclear cells (PBMCs)</u> were isolated on Ficoll–Hypaque density gradients (Biochrom AG) from 5 ml of heparinized venous blood drawn from healthy volunteer donors.

Lentiviral transduction and enrichment of PDX ALL cells

PDX ALL-265 and ALL-199 were established as described (6); Lentiviruses were produced and PDX cells genetically engineered as described (7, 8) and were titrated such that transduction efficiencies were below 1%, to achieve a single genomic integration per cell. As unique variance and to save resources, lentivirally transduced PDX cells were kept in culture to await fluorochrome marker expression; on day 4-6, successfully transduced cells were enriched by flow cytometry using a BD FACSAria^{TM} III cell sorter (Becton-Dickinson) and injected into mice for amplification, as highly transgenic population.

Isolation of transgene-expressing cell populations from murine bone marrow or spleen was performed as described (6). Based on fluorochrome markers, viable cells were either enriched for further experiments or analyzed using the LSRII flow cytometer (Becton-Dickinson).

PDX *in vivo* experiments

PDX *in vivo* experiments were performed and monitored by bioluminescence *in vivo* imaging as previously described (6). To induce KLF4 transgene expression, Doxycyline (DOX) was solved at 1 mg/ml in drinking water containing 5% Glucose (Sigma-Aldrich) and served to mice at free disposition (wtKLF4 or mutKLF4); DOX-containing drinking water was changed weekly.

For pairwise competitive *in vivo* experiments, PDX-ALL cells expressing mock and wtKLF4 (wtKLF4 mixture) or mock and mutKLF4 (control mixture) were mixed at a ratio of 1:1 and injected into the tail vein of NSG mice.

In vivo chemotherapy treatment: PDX ALL bearing mice were treated with PBS or a combination of vincristine (VCR; Merck, Darmstadt, Germany; given once per week on Fridays) and cyclophosphamide (Cyclo; Baxter, USA; given once per week on Mondays). In low dose regimens, mice received 0.2 mg/kg VCR plus 35 mg/kg Cyclo until the experimental endpoint; in high dose regimens, mice received 0.25 mg/kg VCR + 80 mg/kg Cyclo.

In vivo homing assay**:** Mice were injected with PDX-265 cells transduced with mock, mutKLF4 or wtKLF4 constructs; at day 30, when tumor burden was high, DOX was administered to induce transgene expression. 2 days later, PDX cells were harvested from the spleen and mCherry-positive cells were sorted using BD FACSAria^{TM} III. $5x10^5$ PDX-265 cells each were mixed at 1:1 ratio and reinjected into NSG recipients. 3 days after injection, mice were sacrificed and bone marrow cells were isolated. PDX-265 cells were enriched by Mouse cell depletion kit (Miltenyi Biotec, Bergisch Gladbach, Germany) as previously described (8) and the percentage of mock and wtKLF4/mutKLF4 analyzed by flow cytometry.

***In vitro* drug treatment**

For *in vitro* drug treatment, PDX cells or NALM-6 cells were cultured and dilutions in PBS from either Vincristine (1mg/ml stock), APTO-253 (10 mM stock in DMSO, HY-16291, MedChem Express, New Jersey, USA) or Azacitidine (10 mM stock in DMSO, Biozol Diagnostica, Eching, Germany) added to the medium and cells were analyzed 2 days after treatment.

Analysis of cell viability and apoptosis

Cell viability was determined by flow cytometry based on FSC/SSC gating and/or staining with Annexin V. Viability of EBV-infected B cells was measured as previously described (9). In brief, EBV-infected cells were washed twice with FACS staining buffer (PBS, 0.5 % BSA, 2 mM EDTA), resuspended in 200 µl Annexin V staining buffer together with 2 µl Cy5-coupled Annexin V (BioVision, Milpitas, USA) and incubated in the dark for 10 min. Percentage of Annexin V-positive cells was determined using a BD FACS Fortessa instrument (Becton-Dickinson).

Cell cycle analysis

BrdU staining: Per time point, $5x10^5$ EBV-infected cells were seeded initially and cultivated further. Prior to harvest, the cells were incubated with BrdU (10 µM final concentration) and incubated in the dark at 37°C for 1 hour. After BrdU incorporation, the cells were washed with FACS staining buffer and resuspended in Cytofix/Cytoperm (Becton-Dickinson) buffer followed by a 15-20 min incubation on ice. The cells were washed with FACS staining buffer and frozen in freezing medium (90 % FCS, 10 % DMSO). The samples were stored at -80°C for further analysis. The BrdU incorporation was analyzed using the APC BrdU Flow Kit (#552598, BD Pharmingen) according to the manufacturer's recommendations. The cells were thawed, re-fixated with BD Cytofix/Cytoperm buffer for 5 min on ice, washed with BD Perm/Wash buffer, and resuspended in PBS and DNase. The mix was incubated at 37°C for 1 h, then washed with BD Perm/Wash buffer, resuspended in BD Perm/Wash buffer and stained with 1 µl anti-BrdU-APC coupled antibody for 20 min at RT. Afterwards, the cells were washed with BD Perm/Wash buffer, resuspended in 7-AAD solution (20 µl per sample of a 50 µg/ml stock solution, (#420404 Biolegend, San Diego, USA) and FACS staining buffer. After 10 min incubation, the samples were measured with a BD FACS Fortessa instrument and analyzed with the FlowJo software (Tree Star Software, Ashland, USA) to determine the cell cycle distribution.

Propidium Iodide (PI) staining

10^6 viable, mCherry positive sorted cells were fixed in 70% ethanol and stored at -20 ^{o}C overnight. The next day, cells were washed in cold PBS, stained with 50 µg/ml PI

(Sigma-Aldrich) and incubated for 30 min at 37 °C. Afterwards, cells were measured by flow cytometry and analysis was performed using the FlowJo software (Tree Star Software).

Gene expression analysis

Array based expression analysis: The data set of B-ALL (n=306) was used for gene expression analysis (GSE66006, GSE78132, GSE37642) (10-12). Details of the diagnostic work-up, sample preparation, hybridization, image acquisition and analysis have been described previously (10). In brief, for probes to probe set annotation, we used custom chip definition files (13). The HG-U133 A, B chips and HG-U133 Plus 2.0 chips were normalized separately by the robust multichip average method as described (14) and only probes present on all chips were included in the analysis (n=17,389). The batch effect resulting from the use of different chip designs was corrected by applying an empirical Bayesian method as described elsewhere (15).

SCRB sequencing: PDX-265 mock- (n=6), mutKLF4- (n=6) and wtKLF4-expressing cells (n=12) under Dox induction were isolated from mice and cell populations were sorted into lysis buffer composed of 0.2 % Triton X-100 (Sigma-Aldrich, St. Louis, USA) and 2 U/µl of RNAse Inhibitor (Life Technologies). Library preparation followed the recently published single-cell RNA barcoding and sequencing (SCRB-seq) protocol (16) and were sequenced on a HiSeq 1500 instrument (Illumina) as 50 bp single reads. Genes with less than one count per million (cpm) in more than three samples were excluded from the analysis.

Gene expression data were analyzed with the statistical software R (version 3.6.2) (17) and the R-package edgeR (3.28.1). Raw count data were normalized to log-counts per million (CPM) values. Genes described as KLF4 target genes in Ref. (18) were analyzed for differential expression between mock and wtKLF4. Nine genes were not measured by SCRB-Seq, and two genes (Nanog and Lefty1) were not expressed in the sample. The remaining 30 genes were tested with a two-sided Mann-Whitney U test.

Sequencing and data analysis of primary ALL patient samples at diagnosis and 33 days following therapy was described previously; at MRD, the analyzed cells consisted

of highly enriched ALL cells, distinguished from normal bone marrow cells by individualized immune surface markers (6).

RNA-seq based gene expression analysis at early time points following EBV infection: A detailed description of the time course experiment has recently been published by Mrozek-Gorska et al. (19). Raw data are available at the following link (https://www.ebi.ac.uk/arrayexpress/experiments/E-MTAB-7805/) and processed data can be freely accessed online (http://ebv-b.helmholtz-muenchen.de/).

RT-PCR*:* Total RNA was extracted from 10^6 cells using the Qiagen RNeasy Kit (Hilden, Germany) according to manufacturer's instructions. 100 ng RNA was reverse transcribed using iScript cDNA (Bio-Rad). cDNA and primers (Table S4, Eurofins MWG-Operon) were mixed with PCR Master Mix (Thermo Scientific , Waltham, USA) and amplified in 25 cycles (30 sec at 94°C, 30 sec at 60°C, 30 sec at 72°C). Amplified cDNA was loaded on a 2% agarose gel (Invitrogen, Carlsbad, USA) and bands were visualized following staining with Midori^{Green} Advance (Nippon Genetics, Düren, Germany) using the gel imaging system E Box CX5 (Vilber Lourmat, Marne La Vallée, France).

Statistical Analysis

Statistical significance was determined using the "R" software package. Mean values were compared by Student's t-test or analysis of variance, depending on the number of groups and days to be analyzed.

Supplementary References

1. Zuber J, McJunkin K, Fellmann C, Dow LE, Taylor MJ, Hannon GJ, et al. Toolkit for evaluating genes required for proliferation and survival using tetracycline-regulated RNAi. Nat Biotechnol. 2011;29(1):79-83.

2. Liu Y, Zheng B, Zhang XH, Nie CJ, Li YH, Wen JK. Localization and function of KLF4 in cytoplasm of vascular smooth muscle cell. Biochemical and biophysical research communications. 2013;436(2):162-8.

3. Pich D, Mrozek-Gorska P, Bouvet M, Sugimoto A, Akidil E, Grundhoff A, et al. First Days in the Life of Naive Human B Lymphocytes Infected with Epstein-Barr Virus. mBio. 2019;10(5):e01723-19.

4. Delecluse HJ, Hilsendegen T, Pich D, Zeidler R, Hammerschmidt W. Propagation and recovery of intact, infectious Epstein-Barr virus from prokaryotic to human cells. Proc Natl Acad Sci U S A. 1998;95(14):8245-50.

5. Danisch S, Slabik C, Cornelius A, Albanese M, Tagawa T, Chen YA, et al. Spatiotemporally Skewed Activation of Programmed Cell Death Receptor 1-Positive T Cells after Epstein-Barr Virus Infection and Tumor Development in Long-Term Fully Humanized Mice. Am J Pathol. 2019;189(3):521-39.

6. Ebinger S, Ozdemir EZ, Ziegenhain C, Tiedt S, Castro Alves C, Grunert M, et al. Characterization of Rare, Dormant, and Therapy-Resistant Cells in Acute Lymphoblastic Leukemia. Cancer cell. 2016;30(6):849-62.

7. Terziyska N, Castro Alves C, Groiss V, Schneider K, Farkasova K, Ogris M, et al. In vivo imaging enables high resolution preclinical trials on patients' leukemia cells growing in mice. PloS one. 2012;7(12):e52798.

8. Ebinger S, Zeller C, Carlet M, Senft D, Bagnoli JW, Liu W-H, et al. Plasticity in growth behavior of patients' acute myeloid leukemia stem cells growing in mice. Haematologica. 2020:haematol.2019.226282.

9. Steinbruck L, Gustems M, Medele S, Schulz TF, Lutter D, Hammerschmidt W. K1 and K15 of Kaposi's Sarcoma-Associated Herpesvirus Are Partial Functional Homologues of Latent Membrane Protein 2A of Epstein-Barr Virus. Journal of virology. 2015;89(14):7248-61.

10. Herold T, Schneider S, Metzeler KH, Neumann M, Hartmann L, Roberts KG, et al. Adults with Philadelphia chromosome-like acute lymphoblastic leukemia frequently have IGH-CRLF2 and JAK2 mutations, persistence of minimal residual disease and poor prognosis. Haematologica. 2017;102(1):130-8.

11. Fransecky L, Neumann M, Heesch S, Schlee C, Ortiz-Tanchez J, Heller S, et al. Silencing of GATA3 defines a novel stem cell-like subgroup of ETP-ALL. Journal of hematology & oncology. 2016;9(1):95.

12. Herold T, Jurinovic V, Batcha AMN, Bamopoulos SA, Rothenberg-Thurley M, Ksienzyk B, et al. A 29-gene and cytogenetic score for the prediction of resistance to induction treatment in acute myeloid leukemia. Haematologica. 2018;103(3):456-65.

13. Ferrari F, Bortoluzzi S, Coppe A, Sirota A, Safran M, Shmoish M, et al. Novel definition files for human GeneChips based on GeneAnnot. BMC Bioinformatics. 2007;8:446.

14. Irizarry RA, Hobbs B, Collin F, Beazer-Barclay YD, Antonellis KJ, Scherf U, et al. Exploration, normalization, and summaries of high density oligonucleotide array probe level data. Biostatistics. 2003;4(2):249-64.

15. Herold T, Metzeler KH, Vosberg S, Hartmann L, Rollig C, Stolzel F, et al. Isolated trisomy 13 defines a homogeneous AML subgroup with high frequency of mutations in spliceosome genes and poor prognosis. Blood. 2014;124(8):1304-11.

16. Bagnoli JW, Ziegenhain C, Janjic A, Wange LE, Vieth B, Parekh S, et al. Sensitive and powerful single-cell RNA sequencing using mcSCRB-seq. Nat Commun. 2018;9(1):2937.

17. R Core Team (2019). R: A language and environment for statistical computing. 2019 [R Foundation for Statistical Computing, Vienna, Austria.]. Available from: https://www.R-project.org/.

18. Park CS, Shen Y, Lewis A, Lacorazza HD. Role of the reprogramming factor KLF4 in blood formation. J Leukoc Biol. 2016;99(5):673-85.

19. Mrozek-Gorska P, Buschle A, Pich D, Schwarzmayr T, Fechtner R, Scialdone A, et al. Epstein–Barr virus reprograms human B lymphocytes immediately in the prelatent phase of infection. PNAS. 2019;116(32):16046.

Supplementary Tables

Table S1. Clinical characteristics of ALL patients

sample	disease stage*	age [years]	sex	cytogenetics	mutations and gene fusions∞	prior treatment	time (months)#
**ALL-199**	R2	8	f	somatic trisomy21; leukemic homozygous 9p deletion	P2RY8-CRLF2	CoALL 0703 ALL-REZ BFM 2002, S2	0,2
**ALL-265**	R1	5	f	hyperdipoidy with additional 6,13,14,17,18,21,X chromosome	KMT2D, HERC1	CoALL 0703	14

*when the primary sample was obtained; ∞ mutations determined by panel sequencing for 100 genes recurrently mutated in ALL; #time between collection of the sample and death of the patient; R1 = 1st relapse; R2 = 2nd relapse; f = female; N.D. not determined.

see also Ebinger et al., Cancer Cell 2016

Table S2. Gene expression profile comparing KLF4 target genes between mock and wtKLF4

Gene	*p*-value	Mean expression in mock	Mean expression in wtKLF4
**p21**	**0.00075**	**5.82**	**6.54**
p27	0.55	6.67	6.51
p57	0.19	0.36	0.93
Cyclin B1	0.053	6.63	7.01
Cyclin D1	0.92	0.87	0.77
Cyclin D2	0.0069	6.99	7.59
Cyclin E	0.21	2.83	3.39
CDK1	0.25	7.66	7.27
ODC1	0.083	6.14	6.36
IFITM3	0.21	6.04	5.72
p53	0.62	7.37	7.42
Rb	0.75	7.91	7.95
FOXM1	0.34	2.1	2.33
Bax	0.68	3.45	3.55
Bcl2	0.82	7.28	7.2
Noxa	0.00075	8.53	9
Puma	0.62	1.83	1.78
Mcl-1	0.81	1.03	0.89
BIRC5	0.018	7.67	7.92
RANKL	0.56	-0.19	-0.14
NOTCH1	0.013	4.52	5.41
Dmp1	0.56	-0.19	-0.13
SM α-actin	0.84	0.07	0.19
Laminin α 3A	0.65	0.52	0.28
Laminin 1	0.75	5.61	5.46
SM22α	0.079	0.62	0.01
LDHA	0.55	8.98	9.02
PFKP	0.0047	5.39	4.81
hTERT	0.25	2.2	2.66
Oct4	0.072	0.17	-0.15

From Dox-treated mice, PDX ALL-265 cells were isolated; mock transgenic and wtKLF4-expressing cells were subjected to scrb sequencing to obtain gene expression profiles; differential gene expression was tested using a two-sided Mann-Whitney U test.

Table S3. sgRNA sequences

	sgRNA sequence
Gaussia luciferase (GLuc)	GGACTCTTTGTCGCCTTCGT
KLF4 seq#1	GTGGTGGCGCCCTACAACGG
KLF4 seq#2	GAGCGATACTCACGTTATTCG
KLF4 seq#3	GTCGGTCATCAGCGTCAGCAA

Table S4. RT-PCR primer sequence

name	Primer sequences
GAPDH	forward: GAAGGTGAAGGTCGGAGTC
	reverse: GAAGATGGTGATGGGATTTC
KLF4	forward: CGGGCTGATGGGCAAGTT
	reverse: GGGCAGGAAGGATGGGTAA

Supplementary Figures

A semi-dry Western blot

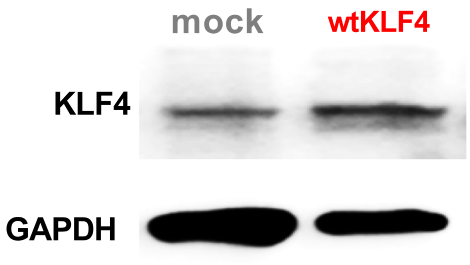

B capillary immunoassay

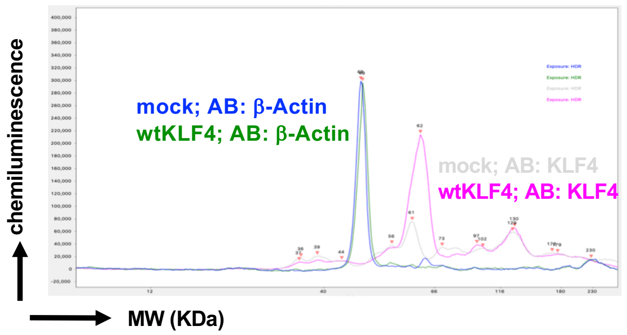

	KLF4 area	GAPDH area	KLF4 expression per GAPDH	Fold change
mock	80241.037	89230.279	0.899257941	1
wtKLF4	101272.16	58313.924	1.736671982	1.931228

	KLF4 area	β-Actin area	KLF4 expression per β-Actin	Fold change
mock	679022	2557381	0.265514603	1
wtKLF4	3717455	2977408	1.248554111	4.702393

semi-dry Western blot

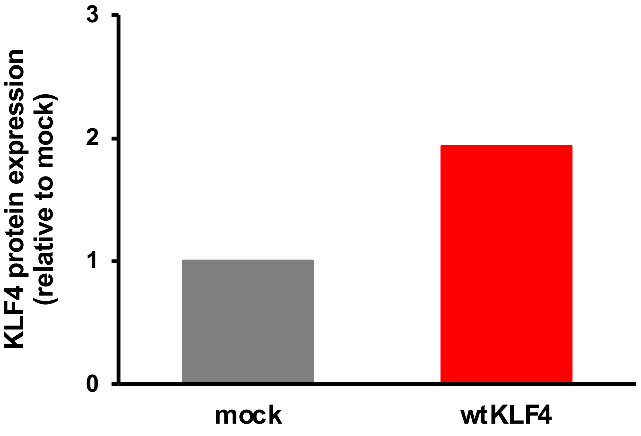

capillary immunoassay

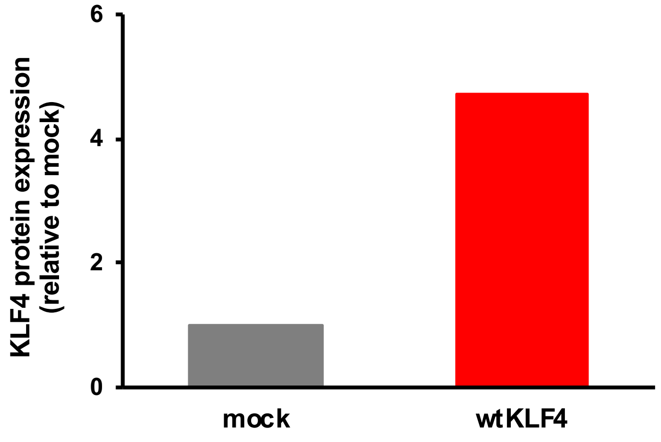

Figure S1 The capillary immunoassay compared to conventional Western Blotting

KLF4 protein level was analyzed in cell lysates from mock-expressing and wtKLF4-expressing cells using two different antibodies and

A. conventional Western Blot, semi-dry (left panels): 50 µg protein lysate from mock or KLF4-expressing cells were loaded analyzed by semi-dry western blot. Band intensities of the resulting blot were quantified using ImageJ 2.0 software. KLF4 expression data was normalized to the loading control, GAPDH, and and values for mock were set to 1 as indicated in the table and depicted in the bar graph.

B. capillary immunoassay (right panels): 2.5 µg protein lysate from mock or KLF4-expressing cells were analyzed by capillary immunoassay. Measured raw data result in histograms from which the Compass for SW Software (version 4.0) calculates the area under the peak and normalized expression levels, using the loading control (β-Actin) for comparison. Fold change relative to mock is indicated in the table and depicted in the bar graph.

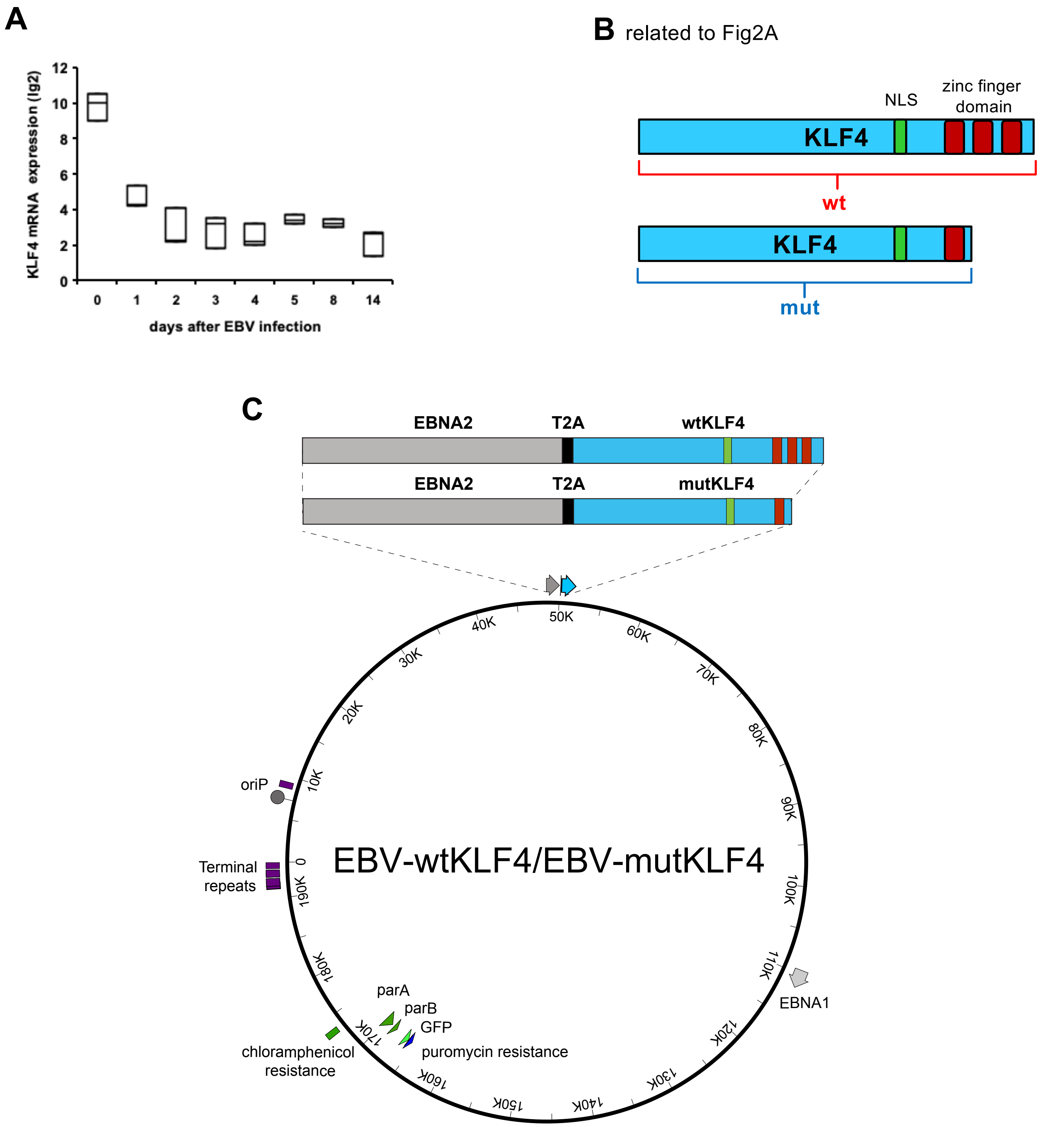

Figure S2 KLF4 mRNA expression and KLF4 expression constructs

A. Primary normal B-cells were infected with EBV as in printed Figure 1 and kinetic extended to 14 days; KLF4 mRNA was measured at the time points indicated as described in Mrozek-Gorska et al. 2019; mean ± SD of 3 independent experiments is shown.

B. Scheme of KLF4: At the carboxy terminus, the KLF4 protein contains 3 zink finger domains (red blocks) which are responsible for DNA binding at GC-rich sequences; the nuclear localization signal (NLS) is indicated by a green box. A truncated mutKLF4 version was generated lacking the two of the three C-terminal zinc finger domains.

C. Scheme of the EBV genome modified to express wtKLF4 or mutKLF4. KLF4 cDNA was linked to the viral EBNA2 sequence via the self-cleaving 2A sequence (T2A); expression of either wtKLF4 or mutKLF4 was driven from the endogenous Wp promoter which also drives EBNA2.

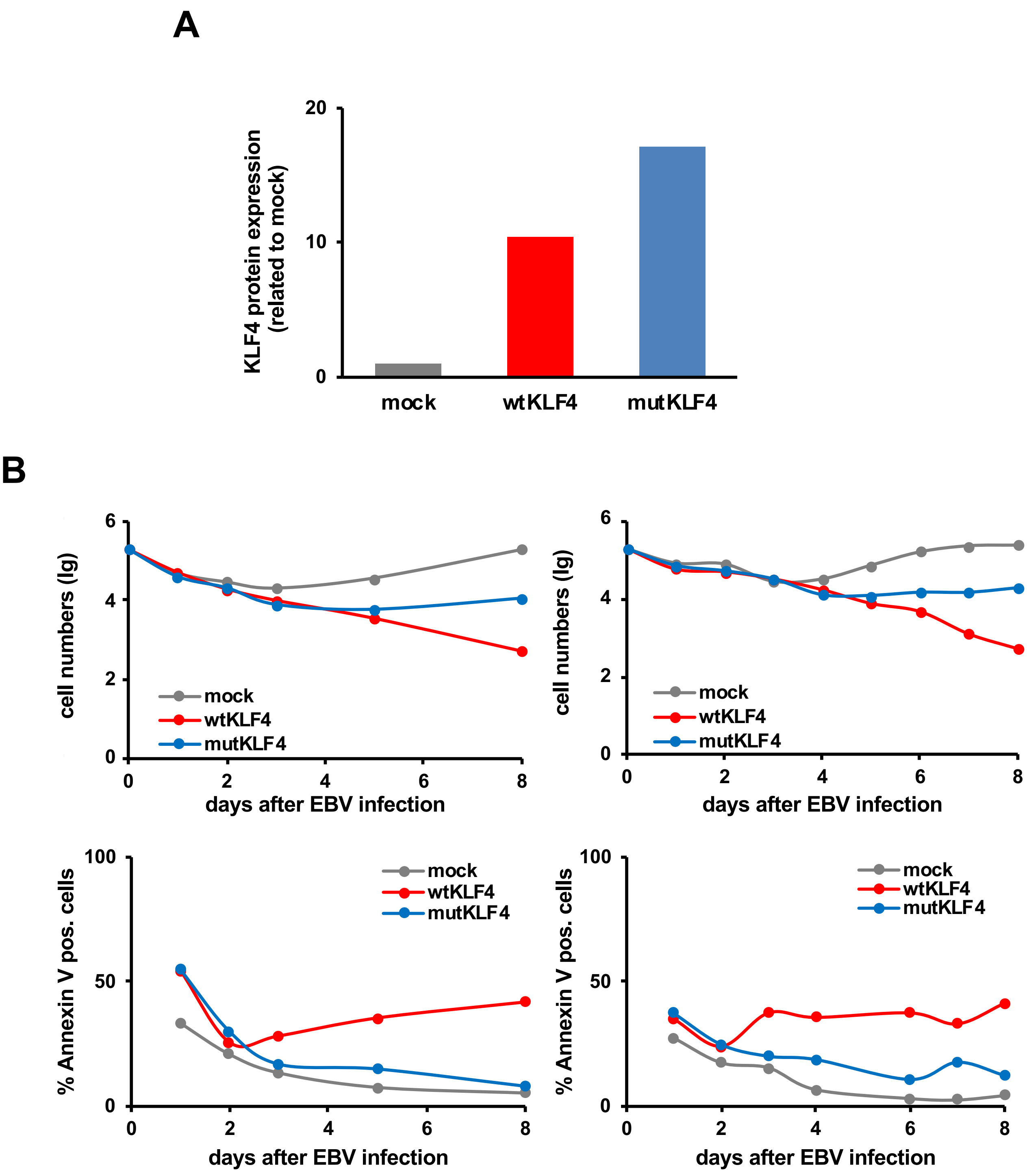

Figure S3 Ectopic expression of KLF4 blocks EBV-mediated oncogenesis

Experiments were performed, analyzed and depicted identically as in printed Figure 1, except that cells from a second (A) or a second and third (B) independent donor were used.

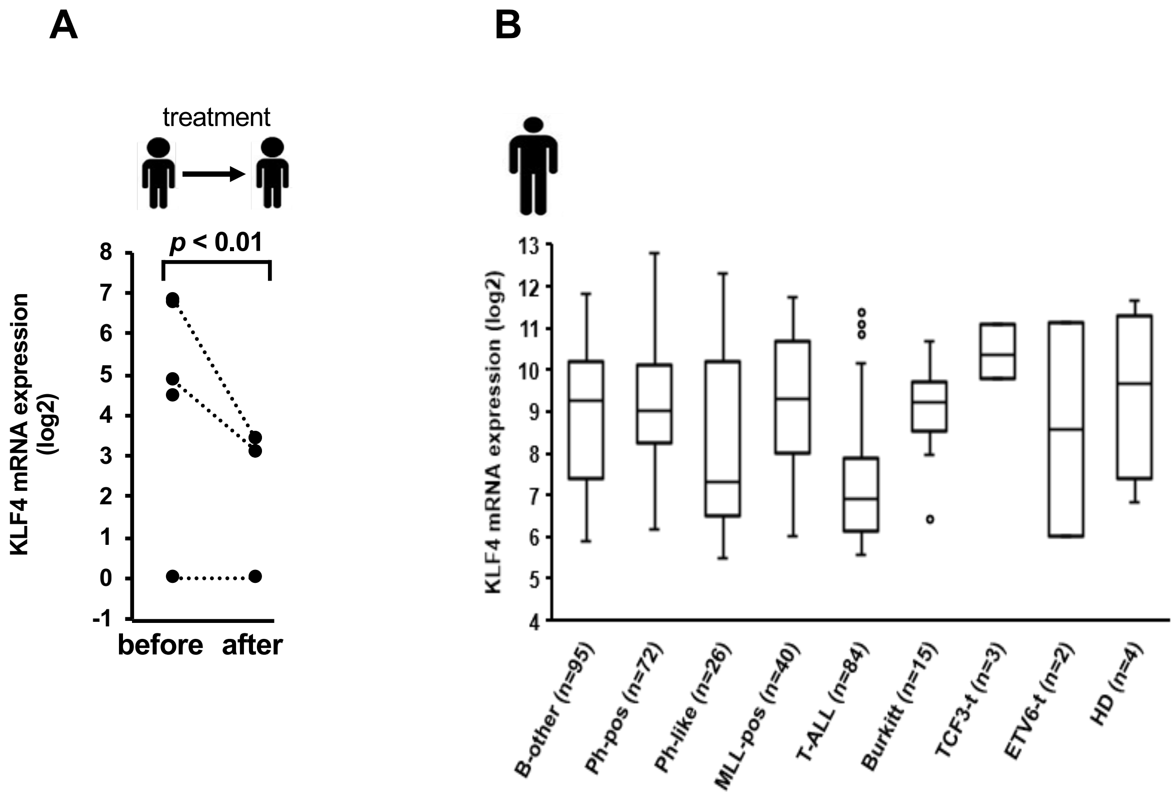

Figure S4 KLF4 mRNA is downregulated in primary patient leukemia cells

A. Expression of KLF4 mRNA in primary samples from pediatric ALL patients at diagnosis (n=5, before) and after the first block of treatment according to the BFM-2009 protocol (n=3, after, day 33). For details on cell sorting and RNA sequencing see Ebinger *et al*. 2016; RNA-seq data are publicly available (GSE83142). Dashed lines indicate matched samples; $p < 0.01$ by two-tailed unpaired t test.

B. Expression of KLF4 mRNA in bone marrow from 306 ALL patients. Datasets are publicly available (GSE66006, GSE78132, GSE37642). Ph-pos: Philadelphia-positive; HD: Hyperdiploidy.

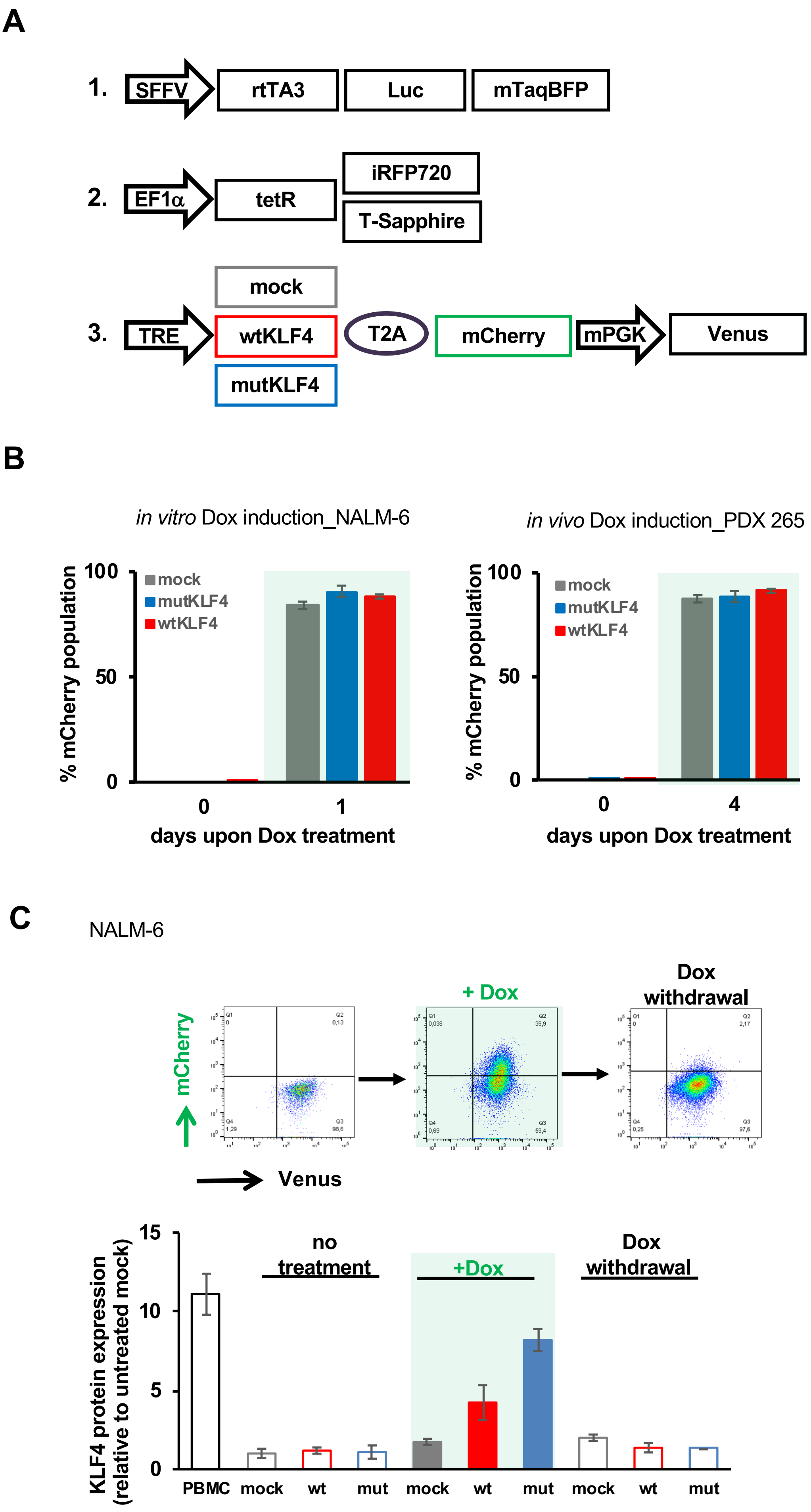
A
1.
SFFV
rtTA3
Luc
mTaqBFP
2.
EF1α
tetR
iRFP720
T-Sapphire
3.
TRE
mock
wtKLF4
mutKLF4
T2A
mCherry
mPGK
Venus
B
in vitro Dox induction_NALM-6
in vivo Dox induction_PDX 265
% mCherry population
100
50
0
mock
mutKLF4
wtKLF4
0
1
4
days upon Dox treatment
C
NALM-6
+ Dox
Dox withdrawal
mCherry
Venus
KLF4 protein expression (relative to untreated mock)
15
10
5
0
no treatment
+Dox
Dox withdrawal
PBMC
mock
wt
mut

Figure S5 A tet-on inducible system to re-express KLF4 in PDX ALL cells

A. Scheme of expression vectors: To enable inducible expression, three constructs were used.

1. The first construct expressed the reverse tet-responsive transactivator 3 (rtTA3) together with Luciferase (Luc) for bioluminescence *in vivo* imaging, and mTaqBFP for enriching transgenic cells by flow cytometry.

2. The second construct expressed the tet repressor (tetR) to reduce leakiness together with either one of two different fluorochromes (iRFP720 or T-Sapphire) to distinguish two different cell populations in a single mouse during competitive *in vivo* assays.

3. The third construct contained either mock, wtKLF4 or mutKLF4, expressed under control of the Dox-inducible tetracycline response element (TRE); a second fluorochrome (mCherry) was directly coupled to KLF4 by a T2A linker sequence, enabling visualization and flow cytometric enrichment of cells successfully expressing the induced transgene upon Dox treatment; in addition, the third expression construct constitutively expressed the fluorochrome Venus for enrichment of transduced cells.

B. <u>High expression levels of mCherry after Dox treatment</u>.
mCherry expression construct is linked to the expression construct of wtKLF4 or mutKLF4 by a T2A site such that mCherry indicates expression of KLF4.

1. Left: 10^6 NALM-6 cells transduced with the indicated constructs were incubated with 0.5µg/ml Dox for 24h and the percentage of mCherry positive cells was determined by flow cytometry. Quantification as mean ± SD of 3 independent experiments is shown.

2. Right: PDX ALL-265 cells transduced with the indicated constructs, enriched by flow cytometry and 60,000 cells injected into NSG mice. Dox was added to the drinking water of mice upon high tumor burden. After 4 days of Dox treatment, mice were sacrificed and percentage of mCherry positive cells from spleen and bone marrow determined by flow cytometry. Quantification as mean ± SD of 3 independent experiments is shown.

C. <u>Reversibility of the tet-on expression system</u>. NALM-6 cells were transduced with mock, wtKLF4 or mutKLF4 constructs and cultured without or with 0.5 µg/ml Dox overnight. Cells were either harvested, or Dox withdrawn and cells cultured for additional 5 days in the absence of Dox. Upper panel: Representative dot plots of NALM-6 cells transduced with the mock construct; expression of mCherry is linked to expression of KLF4 and induced by Dox. Lower panel: KLF4 protein expression was analyzed by capillary immunoassay; β-Actin was used as loading control. KLF4 expression normalized to β-Actin is plotted as fold change relative to PBMC. Quantification as mean ± SD of 3 independent experiments is shown.

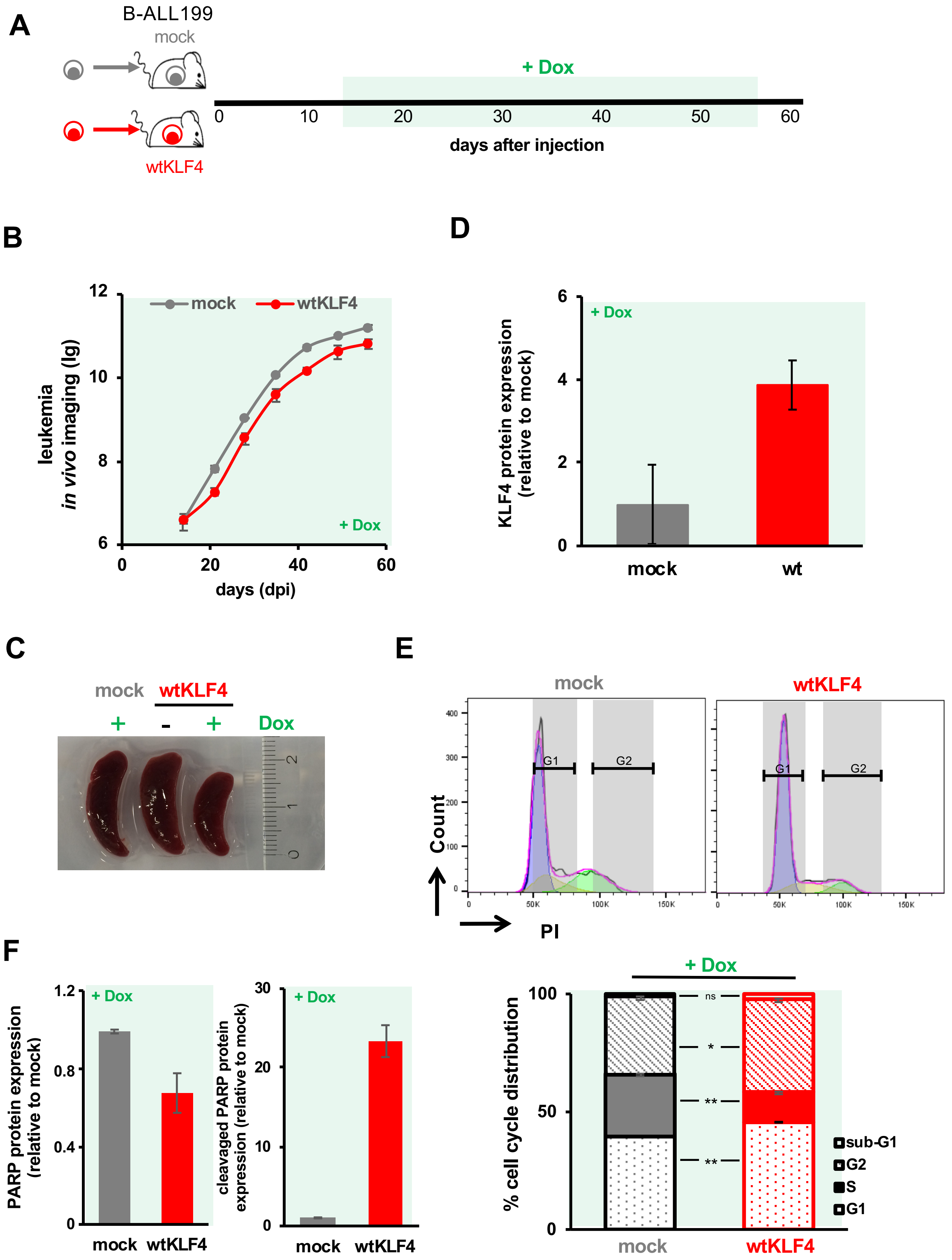
A
B-ALL199
mock
wtKLF4
+ Dox
0 10 20 30 40 50 60
days after injection
B
mock
wtKLF4
leukemia
in vivo imaging (lg)
+ Dox
days (dpi)
D
KLF4 protein expression
(relative to mock)
+ Dox
mock
wt
C
mock
wtKLF4
+ - + Dox
E
mock
wtKLF4
Count
PI
G1
G2
F
PARP protein expression
(relative to mock)
+ Dox
mock wtKLF4
cleaved PARP protein
expression (relative to mock)
+ Dox
mock wtKLF4
+ Dox
% cell cycle distribution
ns
*
**
**
sub-G1
G2
S
G1
mock
wtKLF4

Figure S6 Re-expressing KLF4 inhibits tumor growth through cell cycle arrest and apoptosis in B-ALL PDX cells *in vivo*

Experiments were performed and depicted identically as in printed Figure 3, except that ALL-199 was used instead of ALL-265.

A. ALL-199 cells were injected into the tail vein of 6 NSG mice each. After homing was completed and tumors were established, Dox was added to the drinking water from day 14 (light green background) and leukemia growth monitored by bioluminescence *in vivo* imaging. On day 56 after cell injection, mice were sacrificed, spleens harvested and the successfully Dox-induced, mCherry positive mock or wtKLF4 expressing population enriched by flow cytometry for further analysis.

B. Bioluminescence in vivo imaging after quantification of all 6 mice, depicted as mean ± SEM.

C. Representative spleens of mice, using a KLF4 expressing mouse without Dox treatment for comparison.

D. KLF4 protein level of mCherry positive splenic cells was analyzed by capillary immunoassay; β-Actin served as loading control. Representative analysis from one out of three mice are shown. KLF4 expression of normalized to β-Actin is plotted as fold change relative to mock. Quantification as mean ± SD of 3 independent experiments is shown. .

E. Cell cycle analysis; 10^6 mCherry positive cells were fixed in 70% ethanol, stained with Propidium Iodide (PI) and cell cycle distribution was measured by flow cytometry. Upper panel: Representative histograms of mock (n=3) or wtKLF4-expressing (n=3) cells; lower panel: Quantification as mean ± SD is shown. * $p < 0.05$ ** $p < 0.01$ by two-tailed unpaired t test; ns = not significant.

F. PARP cleavage in mock- (n=3) or wtKLF4-transduced (n=3) PDX ALL-199 cells as determined by caplicary immunoassay; β-Actin was used as loading control. Quantification as mean ± SD is shown.

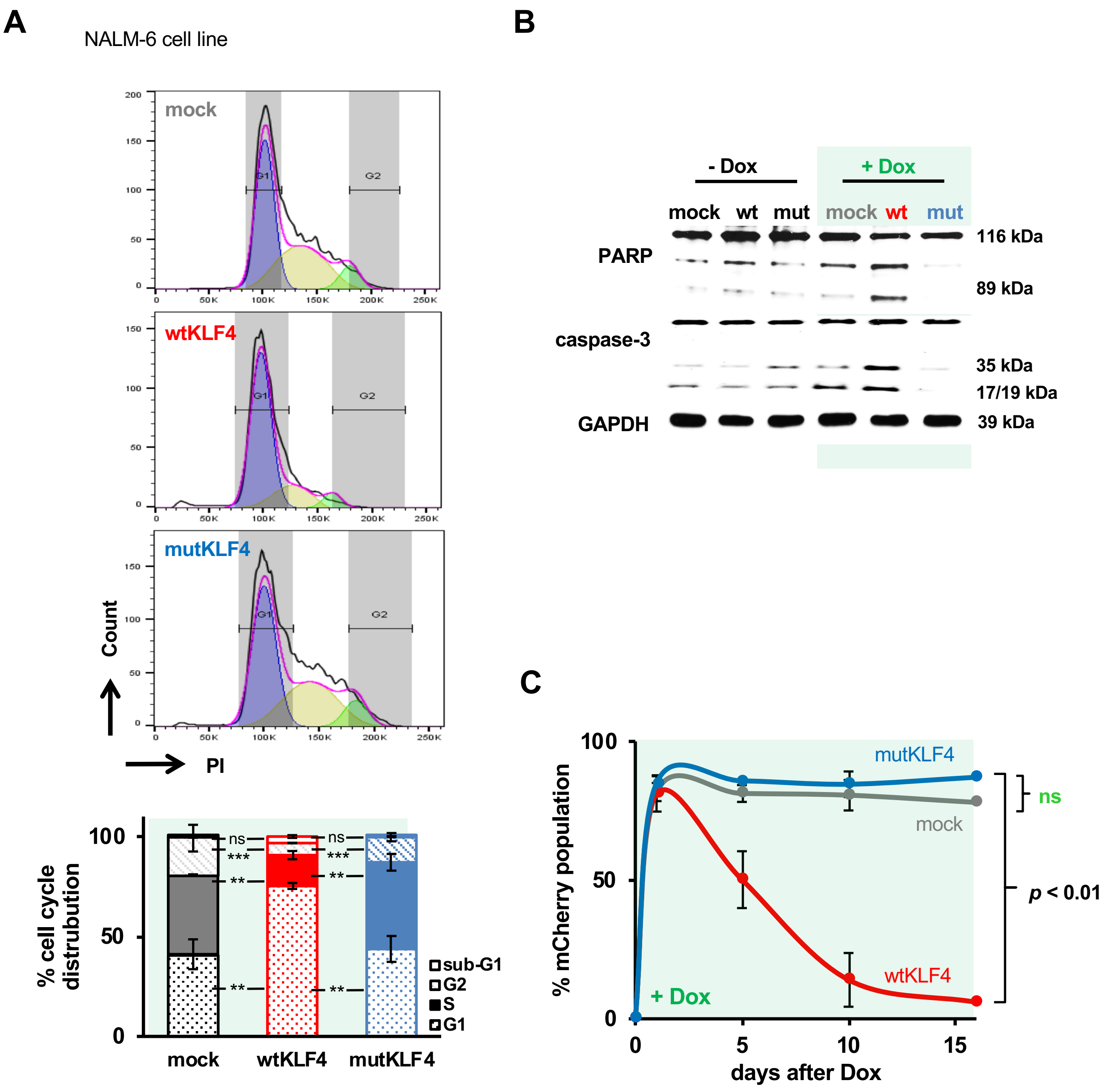

Figure S7 Re-expressing KLF4 inhibits tumor growth through cell cycle arrest and apoptosis in NALM-6 cells *in vitro*

10^6 NALM-6 cells were transduced with the constructs indicated (Figure S5A) and cultured in the presence of 0.5µg/ml Dox.

A. After 2 days, cells were harvested and analyzed and depicted as in Figures 3E and S4E. ns: not significant, ** $p < 0.01$, *** $p < 0.005$ as determined by two-tailed unpaired t test. Quantification as mean ± SD of 3 independent experiments is shown.

B. Western Blot of PARP and caspase-3 was performed as in Figures 3F.

C. mCherry expression indicating expression of mock, wtKLF4 or mutKLF4 was monitored by flow cytometry at the indicated time points; mean ± SD of 3 independent experiments is shown. ns: not significant, $p < 0.01$ as determined by two-tailed unpaired t test.

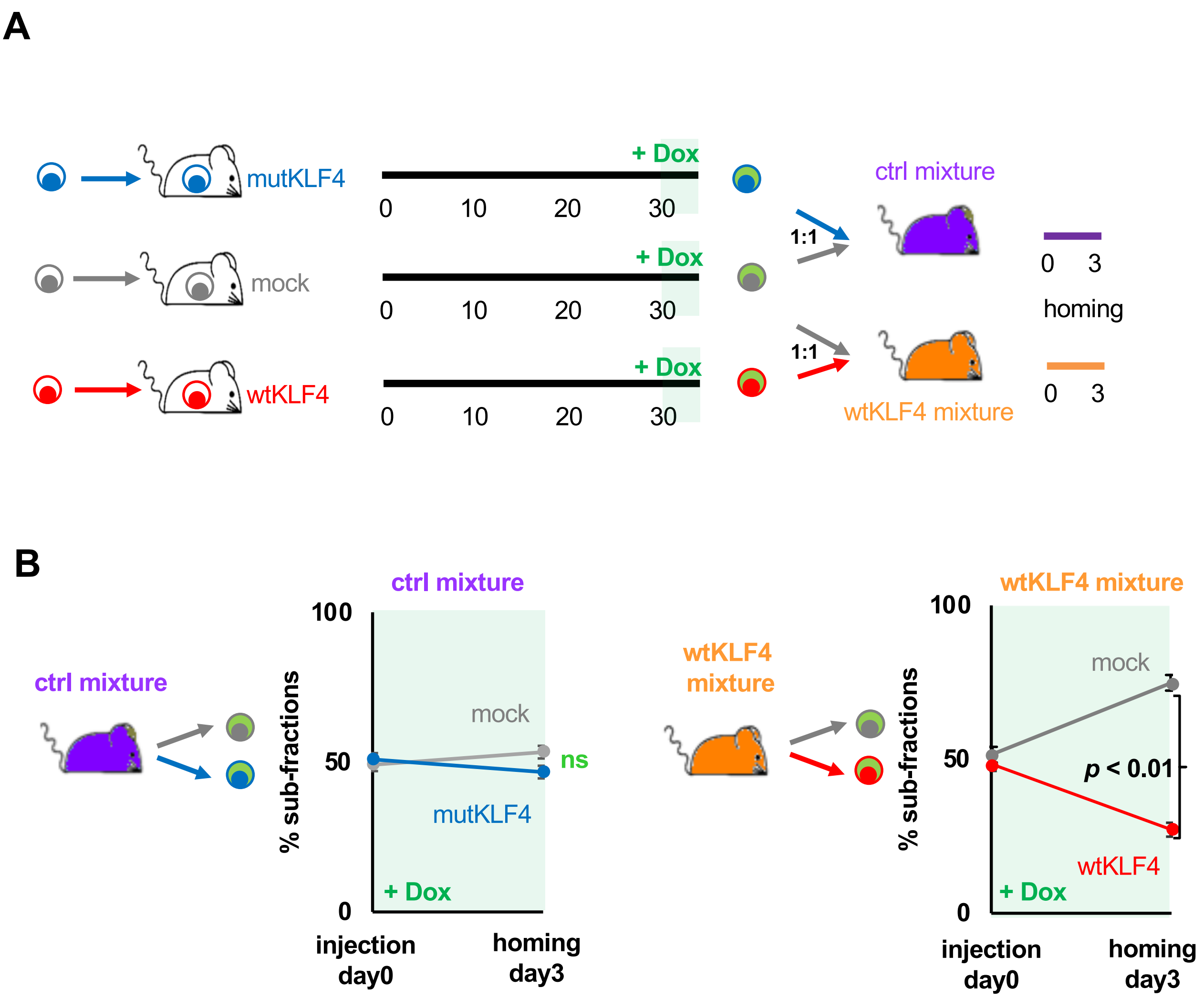

Figure S8 wtKLF4 inhibits homing of PDX ALL cells to the murine bone marrow

A. Experimental procedure: ALL-265 cells transduced with either mock, mutKLF4 or wtKLF4 constructs were separately injected into NSG mice and Dox administered during the last 48 hours before mice were sacrificed. Successfully induced, mCherry positive cells were enriched and mixed into either control or KLF4 mixture, identically as in printed Figures 4 and 5. 10^6 cells of each mixture were re-injected into 3 NSG recipients each. To evaluate homing capacities, bone marrow of secondary recipients was harvested 3 days after injection and analyzed by flow cytometry.

B. Sub-fraction analysis was performed and is depicted as mean ± SEM of 3 mice per group. ns: not significant; $p < 0.01$ as determined by two-tailed unpaired t test.

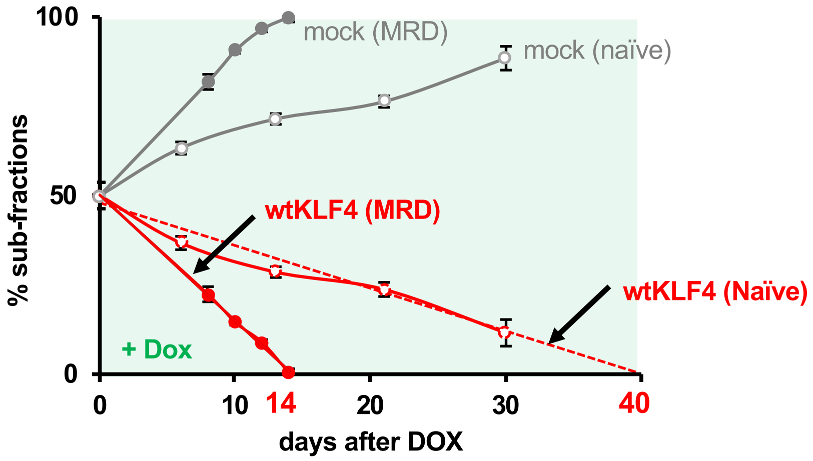

Figure S9 Treatment-surviving cells are especially sensitive towards re-expressing KLF4

Graph compares data from sub-fractions of wtKLF4 mixture from Figure 4B (untreated cells) with Figure 4D (treated cells). Red dashed line represents calculated trend of untreated cells. Quantification as SEM ± SD of 3 mice experiments is shown.

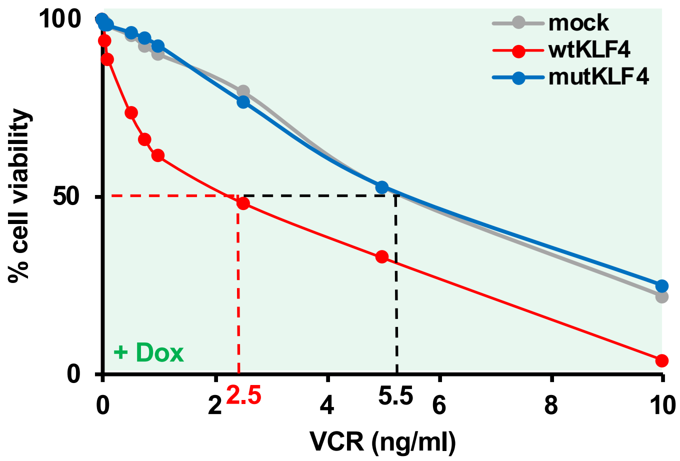

Figure S10 Re-expressing KLF4 sensitizes NALM-6 cells towards Vincristine treatment in vitro

10^6 NALM-6 cells transduced with the indicated constructs were cultured in the presence of 0.5 µg/ml Dox and treated with increasing concentration of Vincristine (VCR) for 48h. Cell viability was analyzed by flow cytometry; all groups were normalized to non-VCR treated cells; mean ± SD of 3 independent experiments is shown. Dashed lines indicates IC50 of mock (black) and wt KLF4 (red) cells.

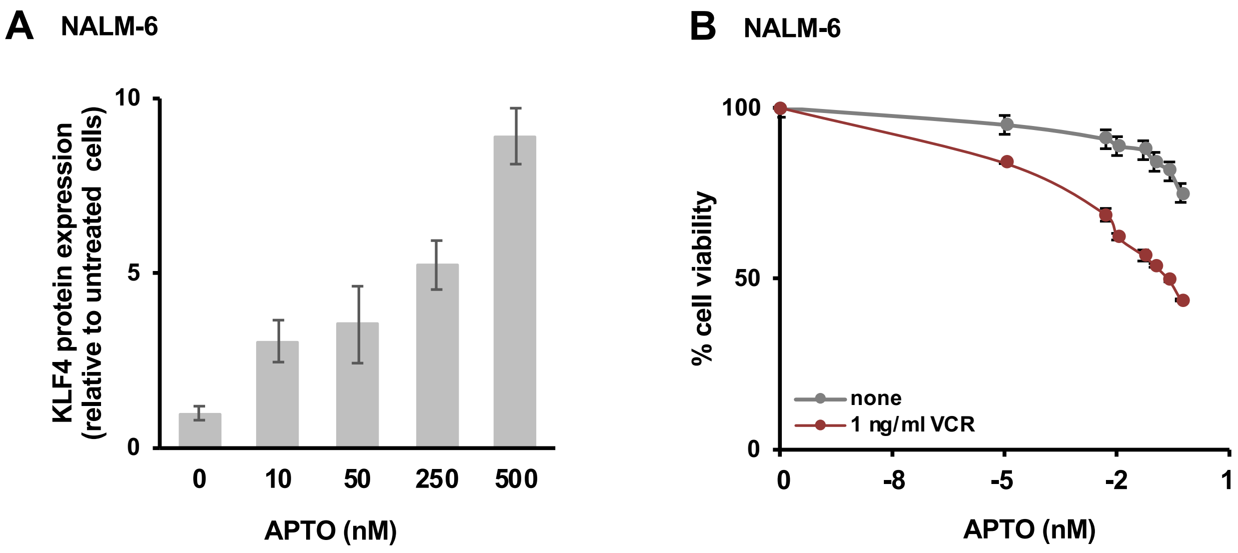

Figure S11 APTO-253 upregulates KLF4 and enhances sensitivity to Vincristine

A. NALM-6 cells were incubated with the indicated concentrations of APTO-253 (APTO) for 3 days and KLF4 protein expression analyzed by capillary immunoassay. Mean ± SD of 3 independent experiments of KLF4 expression normalized to β-Actin is plotted as fold change relative to untreated cells (APTO-253 0nM).

B. NALM-6 cells were incubated with the indicated concentrations of APTO-253 in the presence or absence of 1 ng/ml VCR. After 48h, cell viability was analyzed by flow cytometry; mean ± SD of 3 independent experiments is shown.

A NALM-6 cell line

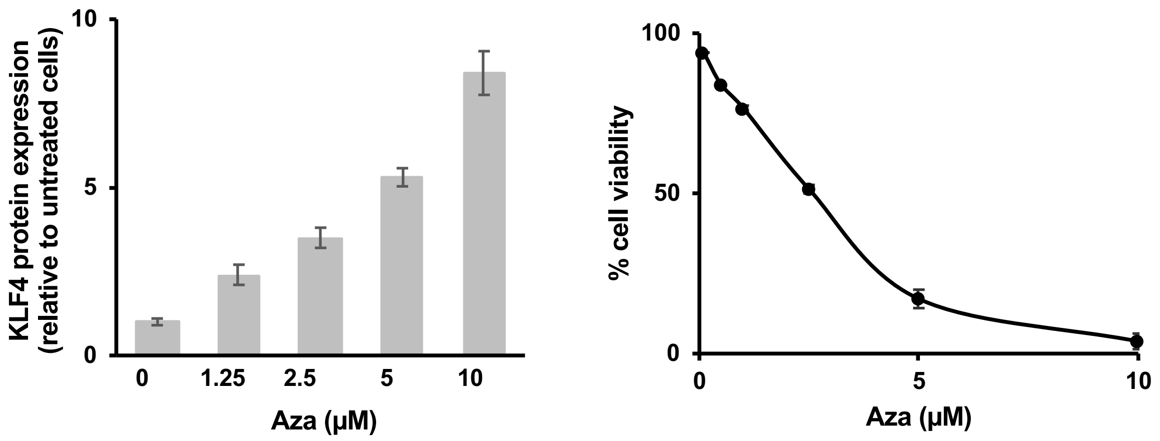

B

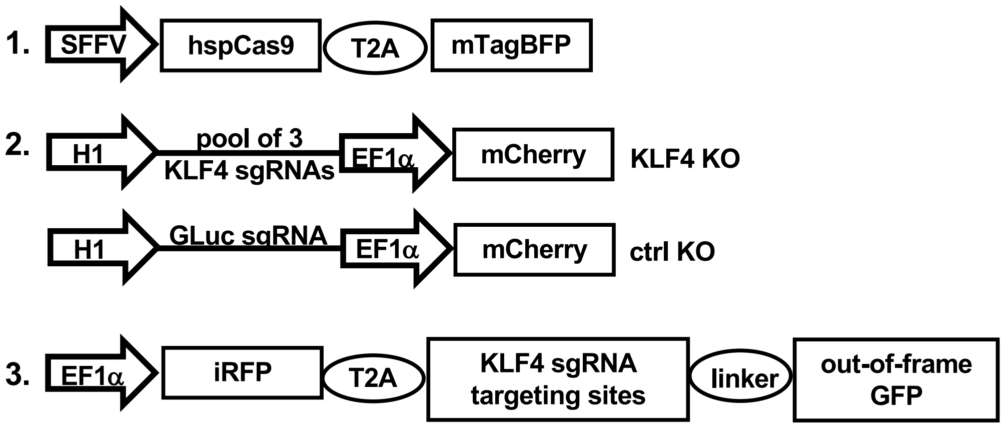

C

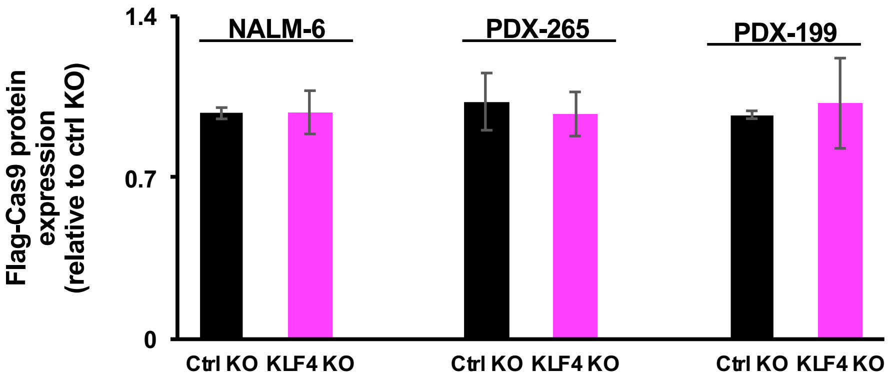

Figure S12 Azacitidine increases expression of KLF4

A. NALM-6 cells were treated with different concentrations of Azacitidine (Aza) for 48h *in vitro.* Left panel: KLF4 protein expression analyzed by capillary immunoassay. KLF4 expression normalized to β-Actin is plotted as fold change relative to untreated control (Aza 0µM). Right panel: Viability was analysed by flow cytometry. Quantification as mean ± SD of 3 independent experiments is shown.

B. Schematic diagram of expression constructs for knockout:

1. A human codon-optimized *S. pyogenes* (hsp) Cas9 was constitutively expressed from the SFFV promoter together with the fluorochrome mTaqBFP, linked via a T2A sequence.

2. The single-guide (sg)RNA was expressed under the control of the H1 Pol III promoter. 3 different KLF4 sgRNAs were cloned individually into the constructs before vectors were pooled for lentiviral transduction. GLuc sgRNA was used as a control. mCherry expression from the EF1α promoter allowed enriching transgenic cells.

3. A reporter construct was used for enriching KLF4 knockout cells; in brief, EF1α drives constitutive expression of iRFP720 as well as out-of-frame GFP 5´ of a PAM-containing Cas9/sgRNA targeting sequences. sgRNA guided gene editing in the targeting sequence by Cas9 shifts GFP into frame so that GFP expressing cells are strongly enriched for KLF4 gene knockout. All the constructs were cloned into the pCDH backbone.

C Cas9 protein expression level was analyzed by capillary immunoassay, normalized to β-Actin and plotted as fold change relative to cells with ctrl KO.

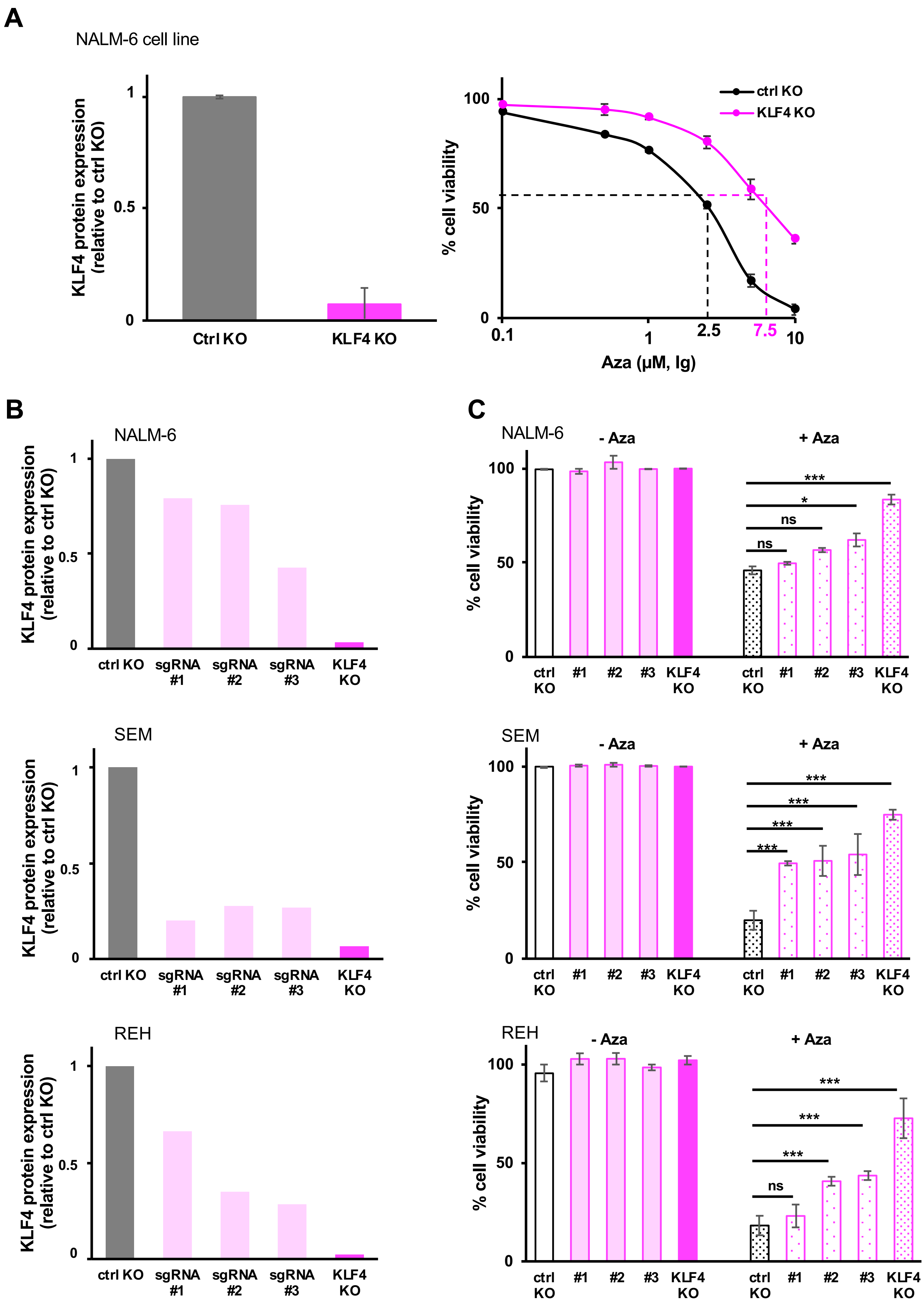
A
NALM-6 cell line
KLF4 protein expression (relative to ctrl KO)
Ctrl KO
KLF4 KO
% cell viability
ctrl KO
KLF4 KO
2.5
7.5
Aza (µM, lg)
B
NALM-6
SEM
REH
KLF4 protein expression (relative to ctrl KO)
ctrl KO
sgRNA #1
sgRNA #2
sgRNA #3
KLF4 KO
C
NALM-6
SEM
REH
- Aza
+ Aza
% cell viability
ns
*
***
ctrl KO
#1
#2
#3
KLF4 KO

Figure S13 Azacitidine-induced cell death depends on upregulating KLF4

A 10^6 KLF4 KO or ctrl KO NALM-6 cells were treated with the indicated concentrations of Aza for 2 days and cell viability was analyzed by flow cytometry. Left panel: KLF4 protein level of NALM-6 cells were detected by capillary immunoassay, KLF4 expression normalized to β-Actin is plotted as fold change relative to Ctrl KO. Right panel: Cell viability was normalized to untreated cells. Dashed lines indicates IC50 of ctrl KO (black) and KLF4 KO (pink) cells. Mean ± SD of 3 independent experiments is shown.

B NALM-6, SEM and REH cells were transduced with the constructs described in Figure S12B, whereby either single sgRNA sequences (#1, #2, #3) or a pool of 3 sgRNAs was transduced. 2-days after transduction, KLF4 protein levels were analysed by capillary immunoassay as described in panel A.

C The sorted-cells were or were not treated with 2.5 μm Aza for 2 days and cell viability analyzed by flow cytometry. Cell viability was normalized to untreated cells. Mean ± SD of 3 independent experiments is shown. * $p < 0.05$, *** $p < 0.005$ by two-tailed unpaired t test; ns = not significant.
